# Pneumococcal Serotype Distribution and Coverage of Existing and Pipeline Pneumococcal Vaccines

**DOI:** 10.1093/infdis/jiaf376

**Published:** 2025-07-22

**Authors:** Laura M King, Kristin L Andrejko, Miwako Kobayashi, Wei Xing, Adam L Cohen, Wesley H Self, J Jackson Resser, Cynthia G Whitney, Adrienne Baughman, Mai Kio, Carlos G Grijalva, Jessica Traenkner, Nadine Rouphael, Joseph A Lewnard

**Affiliations:** School of Public Health, University of California, Berkeley, Berkeley, California, USA; Division of Bacterial Diseases, Centers for Disease Control and Prevention, Atlanta, Georgia, USA; Division of Bacterial Diseases, Centers for Disease Control and Prevention, Atlanta, Georgia, USA; Division of Bacterial Diseases, Centers for Disease Control and Prevention, Atlanta, Georgia, USA; Division of Bacterial Diseases, Centers for Disease Control and Prevention, Atlanta, Georgia, USA; Department of Emergency Medicine, Vanderbilt University Medical Center, Nashville, Tennessee, USA; Department of Biostatistics, Vanderbilt University Medical Center, Nashville, Tennessee, USA; Rollins School of Public Health, Emory University, Atlanta, Georgia, USA; Department of Emergency Medicine, Vanderbilt University Medical Center, Nashville, Tennessee, USA; Department of Medicine, Emory University, Atlanta, Georgia, USA; Department of Health Policy, Vanderbilt University Medical Center, Nashville, Tennessee, USA; Department of Medicine, Emory University, Atlanta, Georgia, USA; Department of Medicine, Emory University, Atlanta, Georgia, USA; School of Public Health, University of California, Berkeley, Berkeley, California, USA

**Keywords:** *Streptococcus pneumoniae*, pneumonia, acute otitis media, invasive pneumococcal disease, pneumococcal conjugate vaccines

## Abstract

**Background:**

Next-generation pneumococcal conjugate vaccines (PCVs) target an expanding array of serotype antigens. We assessed the proportions of invasive pneumococcal disease (IPD) and pneumococcal acute respiratory infections (ARIs) caused by serotypes targeted by existing and pipeline PCVs, and the annual United States pneumococcal disease burdens potentially preventable by these products.

**Methods:**

We estimated serotype distribution and proportions of pneumococcal ARIs (acute otitis media [AOM; children only], sinusitis, nonbacteremic pneumonia) and IPD attributable to serotypes targeted by each PCV using Markov chain Monte Carlo approaches incorporating data from epidemiological studies and Active Bacterial Core surveillance. We then estimated annual numbers of outpatient-managed ARIs, nonbacteremic pneumonia hospitalizations, and IPD cases potentially preventable by PCVs by multiplying disease incidence rates by PCV-targeted disease proportions and vaccine effectiveness estimates.

**Results:**

In children, PCV15, PCV20, PCV24, PCV25, and PCV31 serotypes account for 16% (95% confidence interval, 15%–17%), 31% (30%–32%), 34% (32%–35%), 43% (42%–44%), and 68% (67%–69%) of pneumococcal AOM, respectively. In adults, PCV15, PCV20, PCV21, PCV24, PCV25, and PCV31 serotypes account for 43% (38%–47%), 52% (47%–57%), 69% (64%–73%), 65% (61%–70%), 62% (57%–67%), and 87% (83%–90%) of pneumococcal nonbacteremic pneumonia. For IPD, 42%–85% of pediatric and 42%–94% of adult cases were due to PCV-targeted serotypes. PCV-preventable burdens encompassed 270 000–3 300 000 outpatient-managed ARIs, 2000–17 000 pneumonia hospitalizations, and 3000–14 000 IPD cases annually.

**Conclusions:**

Across pneumococcal conditions, coverage and preventable burdens were lowest for PCV15 and highest for PCV31, with PCV21 also targeting sizeable burdens of adult disease. Comparative estimates of preventable disease burden may inform future policy.

## Teaser

Across pneumococcal conditions, pneumococcal conjugate vaccine (PCV) coverage and preventable burdens were lowest for PCV15 and highest for PCV31, with PCV21 also targeting sizeable burdens of adult disease. Comparative estimates of preventable disease burden may inform future policy.

*Streptococcus pneumoniae* (pneumococcus) causes invasive pneumococcal disease (IPD; bacteremia, meningitis, bacteremic pneumonia) and noninvasive acute respiratory infections (ARIs), including acute otitis media (AOM), sinusitis, and nonbacteremic pneumonia. More than 100 pneumococcal serotypes have been identified via polysaccharide capsule antigens [1], with a minority important in human disease. IPD serotype distribution is well-documented via population- and laboratory-based surveillance [2, 3]. However, little is known about serotype distribution in pneumococcal ARIs, despite their considerable burden among children and adults in the United States (US) [4, 5].

**Figure 1.**
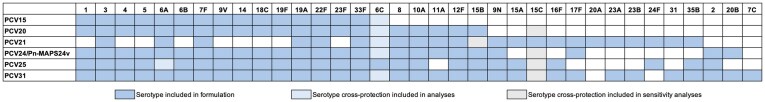
Pneumococcal serotypes targeted by pneumococcal conjugate vaccine (PCV) products. The 24-valent Pn-MAPS24v uses a Multiple Antigen Presenting System platform rather than the traditional conjugate protein platform. PCV24 and Pn-MAPS24v target the same serotypes and so are considered together.

Pneumococcal conjugate vaccines (PCVs) reduce vaccine-serotype colonization and disease [6–8]. In the US, 7-valent PCV (PCV7) was introduced in 2000 followed by 13-valent PCV (PCV13) in 2010. Currently, the Advisory Committee on Immunization Practices (ACIP) recommends 15- and 20-valent PCVs (PCV15, PCV20) for infants [9, 10] and 21-valent PCV (PCV21) alone, PCV20 alone, or PCV15 plus 23-valent pneumococcal polysaccharide vaccine (PPSV23) for PCV-naive adults aged ≥50 years and those aged 19–49 years with certain medical conditions [11, 12]. PCV-induced pressure on vaccine-targeted serotypes results in increased non–vaccine serotype circulation [13–15], necessitating periodic PCV reformulation. At present, 4 pipeline pneumococcal vaccines are undergoing clinical trials: 24-valent PCV (PCV24), 24-valent Pn-MAPS24v (employs Multiple Antigen Presenting System platform; henceforth grouped with PCV24), 25-valent PCV (PCV25), and 31-valent PCV (PCV31) (Figure 1). Vaccine formulations are largely informed by serotypes in IPD; it is unknown to what extent current and pipeline PCVs may contribute to reductions in ARIs.

We aimed to estimate serotype distribution and corresponding PCV-targeted proportions of IPD and noninvasive pneumococcal ARIs in US children and adults using Markov chain Monte Carlo (MCMC) approaches incorporating data from studies of serotype distribution in these syndromes. We used these results to model disease burdens potentially preventable by existing and pipeline PCVs in the US.

## METHODS

We estimated serotype distribution and proportions of noninvasive pneumococcal ARIs (AOM [children only], sinusitis, nonbacteremic pneumonia) and IPD attributable to serotypes targeted by each PCV. We then estimated potential vaccine-preventable burdens by multiplying pneumococcal disease incidence rates by PCV-targeted proportions of disease and vaccine effectiveness (VE) estimates. Supplementary Table 1 summarizes data inputs.

### Serotype Distribution in Pediatric ARIs

We conducted a literature review of studies from the PubMed database (Supplementary Table 2) with data on pneumococcal serotypes in nasopharyngeal samples from children with ARIs in high-income countries after PCV13 implementation. We excluded studies not written in English, those from countries using multiple PCVs, and those aggregating data from children with and without ARI.

We conducted meta-analyses of identified studies (Supplementary Table 3) to estimate serotype distributions among pneumococcal isolates sampled from children with (1) AOM and (2) any ARI. No post-PCV13 studies evaluated sinusitis etiology and only 1 evaluated pneumonia etiology [16]. Thus, parameterization of pneumococcal sinusitis and nonbacteremic pneumonia serotype distributions relied on data from 1 pneumonia study together with AOM studies. We considered all serotypes except 15D [17], only identified in IPD, resulting in 100 serotype categories, including an unencapsulated category. We distinguished counts of nontypeable isolates in primary study data according to whether serotypes were not identified or isolates were determined to lack capsular antigens.

For (1) AOM and (2) other ARIs, we estimated serotype-specific proportions using an MCMC approach drawing on serotype-specific frequencies and sample sizes in each study. We defined p as a vector of serotype-specific prevalences ($p_{i}$ for i∈{1,…,100}) among all pneumococcal isolates, such that $\overset{100}{\underset{i=1}{\sum }}\;p_{i}=1.$ For each study *j*, we defined the vector of serotype-specific frequencies $x_{j}=\{x_{1,j},\;x_{2,j},\ldots ,\;x_{100,j}\}$ as a multinomial draw parameterized by serotype distributions across studies and the sample size $n_{j}=\underset{i}{\sum }\;x_{i,j},\;$


$$x_{j}∼\mathrm{Multinom}(n_{j},\;p).$$

Where studies aggregated data across serotypes/serogroups or did not report all serotypes, we summed corresponding $p_{i,j}$ values to align with the categories represented by the reported $x_{j}$ data. We applied the correction


$$p_{i}=\frac{\;{\hat{\;p}}_{i}}{\sum_{i=1}^{100}\;{\hat{\;p}}_{i}}$$

to the p proposal distribution to ensure that serotype prevalences summed to 1. We conducted 5 000 000 draws with 100 000 draw burn-ins.

### Serotype Distribution in IPD

We used serotype-specific counts from 2015–2019 Active Bacterial Core surveillance (ABCs) data [18] to parameterize IPD serotype distributions. We stratified serotype-specific IPD data by age category (0–17, 18–49, 50–64, and ≥65 years) and sampled serotype distribution vectors via MCMC, parameterized according to multinomial serotype frequencies. For IPD, we included 15D, thus considering 101 serotypes.

### Serotype Distribution in Adult Nonbacteremic Pneumonia

We used serotype-specific counts from the Pneumococcal pNeumonia Epidemiology, Urine serotyping, and Mental Outcomes (PNEUMO) study [19] supplemented with ABCs data to parameterize serotype distributions in adult nonbacteremic pneumococcal pneumonia. The PNEUMO study used a novel serotype-specific urinary antigen detection (SSUAD) assay to identify 30 pneumococcal serotypes: 1, 3, 4, 5, 6A/C, 6B, 7F, 8, 9N, 9V, 10A, 11A, 12F, 14, 15A, 15C, 16F, 17F, 18C, 19A, 19F, 20A, 22F, 23A, 23B, 23F, 24F, 31, 33F, and 35B. In the study population, 20% of patients (69/352) with pneumococcal pneumonia did not test positive for SSUAD-identified serotypes.

For the 30 SSUAD serotypes, we used MCMC to estimate a serotype-specific prevalence vector π subset to SSUAD serotypes from data among the 283 patients with SSUAD-serotype pneumonia. We combined 6A/C due to assay cross-reactivity. For non-SSUAD serotypes, we assumed their distribution resembled that in adult IPD due to non-SSUAD serotypes:


$$\pi_{i}=\frac{\;p_{i}^{\mathrm{IPD}}}{\sum_{i\notin \mathrm{SSUAD}}\;p_{i}^{\mathrm{IPD}}}.$$

We multiplied resulting serotype-specific prevalence estimates by proportions of patients experiencing nonbacteremic pneumococcal pneumonia associated with SSUAD and non-SSUAD serotypes:


$$p_{i\in \mathrm{SSUAD}}=\pi_{i\in \mathrm{SSUAD}}\times \frac{283}{352}$$

$$p_{i\notin \mathrm{SSUAD}}=\pi_{i\notin \mathrm{SSUAD}}\times \frac{69}{352}.$$

### PCV-Targeted Pneumococcal Disease

By condition, we defined coverage for each PCV as the sum of estimated prevalences of targeted serotypes. In primary analyses, we only considered PCV21 in adults due to its adult-only indication [20]. We conducted sensitivity analyses for PCV21-preventable burdens of disease among children at increased risk of pneumococcal disease given current clinical trials [21]. We considered 6A/C together, consistent with previous studies [22], due to demonstrated cross-protection [23, 24] and SSUAD cross-reactivity [19, 25]. We considered 15B/C separately in primary analyses and together in sensitivity analyses. Although vaccination with PCV20 led to detection of anti-15C antibody in adults [26] and children [27], clinically relevant cross-protection is unknown.

### Preventable Disease Burdens

We estimated direct preventable disease burdens as the products of pneumococcal disease incidence, VE estimates, and PCV serotype coverage by condition and age group.

We used multiple sources to estimate the incidence of pneumococcal ARIs and IPD. When possible, we used 2019 data to capture the most recent data without impact from coronavirus disease 2019 (COVID-19) pandemic–associated shifts in disease transmission and healthcare-seeking behavior. For IPD, we obtained incidence estimates from ABCs data [28]. For pneumococcal ARIs, we estimated burdens by multiplying all-cause AOM, pneumonia, and sinusitis incidence rates by published estimates of pneumococcal-attributable proportions of cases (Supplementary Table 4). For nonbacteremic pneumonia, we generated separate estimates by inpatient and outpatient settings. National counts of 2019 inpatient all-cause pneumonia were estimated using National Inpatient Sample [29] data. To obtain nonbacteremic pneumococcal pneumonia hospitalizations, we subtracted estimated bacteremic pneumococcal pneumonia cases from total pneumococcal hospitalization estimates. Bacteremic pneumococcal pneumonia cases were estimated by multiplying national IPD case counts by the proportion that are bacteremic pneumonia (72.1%) [28]. We assumed outpatient-managed pneumonia was nonbacteremic. We estimated AOM and sinusitis burdens using outpatient visit incidence. Estimates of outpatient-managed AOM, pneumonia, and sinusitis were derived from 2016 and 2019 National Ambulatory and National Hospital Ambulatory Medical Care Surveys (NAMCS/NHAMCS) and 2016–2019 Meritage MarketScan Commercial and Medicaid databases following previously described methods [30, 31]. We used the ratio of visit incidence in NAMCS/NHAMCS and MarketScan among adults aged 50–64 years to impute MarketScan incidence rates in adults ≥65 years. Only 2016 and 2019 NAMCS/NHAMCS data were used due to data limitations in 2017–2018 datasets [32]. For outpatient-managed pneumonia, we stratified pediatric age groups as <2 and 2–17 years to ensure adequate sample size for NAMCS/NHAMCS projection validity [32]. We propagated uncertainty by fitting age- and condition-specific burden estimates to Gamma distributions. We multiplied incidence rates by 2019 bridged-race census estimates [33] to obtain national counts.

For age groups (5–17, 18–49 years) for which PCVs are only recommended for individuals at increased risk of pneumococcal disease [9–11, 34], we estimated burdens among (1) all individuals, and (2) those with risk conditions, using published estimates (Supplementary Table 5).

PCV licensure is based on immune response noninferiority; clinical protection estimates are not yet available for PCVs in this study. Age-specific vaccine-serotype VE estimates were extracted from studies of PCV7 and PCV13 for all conditions except pediatric nonbacteremic pneumonia (Supplementary Table 6). Although protective against nonbacteremic pneumonia in children [35], PCV VE against vaccine-serotype disease is uncertain [16, 36]. Consistent with prior work [37], we obtained VE against vaccine-serotype nonbacteremic pneumonia in children by multiplying VE against vaccine-serotype pediatric IPD by the ratio of vaccine-serotype VE estimates in adults for nonbacteremic pneumonia and IPD (45%:75%) [38]. We propagated uncertainty by fitting beta distributions to published estimates.

As prelicensure immunogenicity trials of PCV15/20/21 lacked clinical endpoints, real-world VE has yet to be determined. Data from previous comparative PCV7/13 and PCV10/13 evaluations in children suggest that differences between products in serotype-specific immunogenicity correlate with differences in protection against colonization (Supplementary Figure 1). Due to uncertainty in VE for current and future products, we conducted sensitivity analyses evaluate the impact of increasing or decreasing VE by 10% and 25% for each PCV product.

For preventable burden estimates, we excluded PCV13 serotypes from PCV-targeted proportions of disease, assuming that residual PCV13-serotype disease was not further preventable by higher-valency PCVs. We did not consider indirect effects.

This activity was reviewed by the Centers for Disease Control and Prevention (CDC), was deemed not to represent human subjects research, and was conducted consistent with applicable federal law and CDC policy; see, for example, 45 Code of Federal Regulations (C.F.R.) part 46.102(l)(2), 21 C.F.R. part 56; 42 United States Code (U.S.C.) §241(d); 5 U.S.C. §552a; 44 U.S.C. §3501 et seq.

## RESULTS

### Serotype Distribution

**Figure 2.**
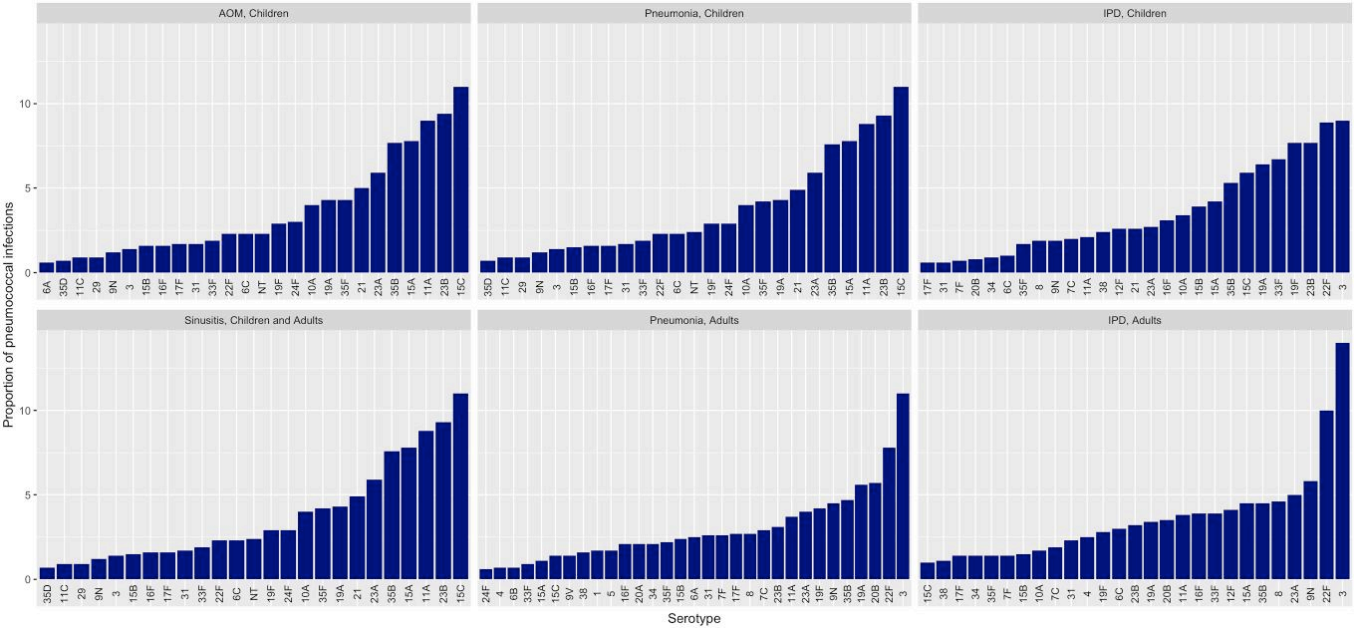
Serotype-specific proportions of pneumococcal infections by condition and age group. Serotypes accounting for <0.05% of pneumococcal isolates within a condition and age group not displayed. All serotype distribution estimates are detailed in Supplementary Table 7. Abbreviations: AOM, acute otitis media; IPD, invasive pneumococcal disease.

Estimated via meta-analysis, the most prevalent serotypes in pediatric pneumococcal ARIs were 15C, 23B, 11A, 15A, 35B, and 23A, together accounting for >50% of isolates (Figure 2; Supplementary Table 7). Serotypes 3, 22F, 20B, 19A, 35B, 9N, 19F, and 23A were the most frequent causes of adult nonbacteremic pneumococcal pneumonia. Serotypes 6A/C were common in adults aged ≥65 years but infrequently identified among adults aged <65 years. Serotype 3 accounted for 1.4% (95% confidence interval [CI], 1.1%–1.7%) of pneumococci in pediatric ARIs and 11.6% (95% CI, 8.7%–15.0%) in adult nonbacteremic pneumonia.

Serotype 3 was the most prevalent serotype in IPD, followed by 22F (Figure 2; Supplementary Table 7). In pediatric IPD, the 10 most prevalent serotypes (3, 22F, 19F, 23B, 33F, 19A, 15C, 35B, 15A, 15B) accounted for almost two-thirds of all cases. In adult IPD, 9N was the third most common serotype, accounting for 5.8% (95% CI, 5.4%–6.2%) of cases.

### PCV Pneumococcal Disease Coverage

In children, PCV15, PCV20, PCV24, PCV25, and PCV31 serotypes accounted for 16.1% (95% CI, 15.2%–17.0%), 30.7% (95% CI, 29.6%–31.8%), 33.6% (95% CI, 32.4%–34.8%), 43.0% (95% CI, 41.8%–44.2%), and 68.0% (95% CI, 66.8%–69.2%) of pneumococcal AOM cases, respectively (Table 1). Serotype distribution estimates for all pediatric ARIs were similar. For pediatric IPD, PCV15 serotypes accounted for 41.7% (95% CI, 38.9%–44.7%) of cases, PCV20, PCV24, and PCV25 serotypes caused 56%–68% of cases, and PCV31 serotypes accounted for 85.0% (95% CI, 82.6%–87.1%) of cases. Additionally, 75.3% (95% CI, 72.6%–77.8%) of pediatric IPD cases were due to serotypes in PCV21 (Supplementary Table 8).

**Table 1.** Proportion of Pneumococcal Infections in Children Aged 0–17 Years Due to Serotypes Targeted by Pneumococcal Conjugate Vaccine Products^a,b,c^

Condition	Percent of Pneumococcal Isolates (95% CI)				
	PCV15	PCV20	PCV24^d^	PCV25	PCV31
AOM	16.1 (15.2–17.0)	30.7 (29.6–31.8)	33.6 (32.4–34.8)	43.0 (41.8–44.2)	68.0 (66.8–69.2)
Sinusitis, pneumonia^e,f^	16.7 (15.9–17.7)	31.2 (30.1–32.4)	34.2 (33.0–35.3)	43.6 (42.4–44.8)	68.2 (67.1–69.4)
IPD	41.7 (38.9–44.7)	55.9 (52.9–58.9)	59.3 (56.4–62.3)	68.2 (65.3–71.0)	85.0 (82.6–87.1)

Abbreviations: AOM, acute otitis media; CI, confidence interval; IPD, invasive pneumococcal disease; PCV, pneumococcal conjugate vaccine.

^a^PCV15 includes serotypes 1, 3, 4, 5, 6A/C, 6B, 7F, 9V, 14, 18C, 19F, 19A, 22F, 23F, 33F. PCV20 includes serotypes 1, 3, 4, 5, 6A/C, 6B, 7F, 8, 9V, 10A, 11A, 12F, 14, 15B, 18C, 19F, 19A, 22F, 23F, 33F. PCV24 includes serotypes 1, 2, 3, 4, 5, 6A/C, 6B, 7F, 8, 9N, 9V, 10A, 11A, 12F, 14, 15B, 17F, 18C, 19F, 19A, 20B, 22F, 23F, 33F. PCV25 includes serotypes 1, 2, 3, 4, 5, 6B, 6A/C, 7F, 8, 9N, 9V, 10A, 12F, 14, 15A, 15B, 16F, 18C, 19F, 19A, 22F, 23F, 24F, 33F, 35B. PCV31 includes serotypes 1, 2, 3, 4, 5, 6A/C, 6B, 7F, 7C, 8, 9N, 9V, 10A, 11A, 12F, 14, 15A, 15B, 16F, 17F, 18C, 19F, 19A, 20B, 22F, 23F, 23A, 23B, 31, 33F, 35B.

^b^Assuming no 15B and 15C cross-protection.

^c^PCV21 not considered as it is intended only for use in adults. Sensitivity analyses considering PCV21 coverage for children with risk-based indications for PCVs presented in Supplementary Table 8.

^d^Also includes Pn-MAPS24v.

^e^Nonbacteremic pneumonia. Bacteremic pneumonia included in IPD definition.

^f^Sinusitis and pneumonia serotype distribution and PCV coverage estimates derived from meta-analysis of serotype distribution in pediatric AOM and pneumonia.

In adults, PCV15 and PCV20 serotypes accounted for 42.6% (95% CI, 37.8%–47.1%) and 52.1% (95% CI, 47.3%–56.7%) of nonbacteremic pneumococcal pneumonia while PCV21, PCV24, and PCV25 serotypes accounted for 62%–69% and PCV31 serotypes accounted for 86.7% (95% CI, 82.6%–89.9%; Table 2). Proportions of cases targeted by PCV15 and PCV20 were higher among adults aged ≥65 years compared with those aged 18–49 years (44%–56% vs 37%–45%). In contrast, PCV21-targeted serotypes were more prevalent among younger adults than older adults. In adult IPD, PCV15, PCV20, PCV21, PCV24, PCV25, and PCV31 targeted 42.3% (95% CI, 41.4%–43.1%), 58.1% (95% CI, 57.2%–58.9%), 82.0% (95% CI, 81.3%–82.7%), 68.8% (95% CI, 68.0%–69.6%), 72.9% (95% CI, 72.2%–73.7%), and 93.9% (95% CI, 93.5%–94.3%) of cases. For all PCVs, except PCV21, IPD coverage was inversely related to age.

**Table 2.** Proportion of Pneumococcal Infections in Adults Aged ≥18 Years Due to Serotypes Targeted by Pneumococcal Conjugate Vaccine Products^a,b^

Condition and Age Group	Percent of Pneumococcal Isolates (95% CI)					
	PCV15	PCV20	PCV21	PCV24^c^	PCV25	PCV31
Sinusitis^d^						
All ≥18 y	16.7 (15.9–17.7)	31.2 (30.1–32.4)	76.7 (75.5–77.9)	34.2 (33.0–35.3)	43.6 (42.4–44.8)	68.2 (67.1–69.4)
Pneumonia^e^						
All ≥18 y	42.6 (37.8–47.1)	52.1 (47.3–56.7)	68.5 (63.6–72.9)	65.4 (60.7–69.9)	61.8 (56.7–66.5)	86.7 (82.6–89.9)
18–49 y	36.5 (26.7–46.5)	45.1 (34.8–55.0)	72.8 (61.4–81.0)	62.8 (52.2–72.2)	60.0 (48.3–69.6)	85.6 (73.2–91.9)
50–64 y	44.0 (36.2–51.2)	52.2 (44.4–59.3)	67.0 (59.2–74.2)	66.9 (59.3–73.9)	61.8 (52.8–68.9)	85.9 (77.8–90.8)
≥65 y	44.3 (36.4–51.8)	56.1 (47.7–63.5)	66.0 (57.4–73.2)	65.2 (57.1–72.7)	62.3 (53.2–69.6)	87.2 (78.6–92.3)
IPD						
All ≥18 y	42.3 (41.4–43.1)	58.1 (57.2–58.9)	82.0 (81.3–82.7)	68.8 (68.0–69.6)	72.9 (72.2–73.7)	93.9 (93.5–94.3)
18–49 y	42.8 (41.0–44.7)	62.1 (60.3–63.9)	79.1 (77.5–80.5)	75.6 (74.0–77.2)	75.3 (73.7–76.9)	95.0 (94.2–95.8)
50–64 y	42.4 (41.3–43.5)	59.2 (57.8–60.6)	81.5 (80.4–82.6)	71.7 (70.4–73.0)	73.5 (72.2–74.8)	94.2 (93.5–94.8)
≥65 y	42.1 (40.8–43.5)	55.0 (53.6–56.3)	83.9 (82.9–84.8)	62.7 (61.4–64.0)	71.1 (69.9–72.3)	93.0 (92.3–93.7)

Abbreviations: CI, confidence interval; IPD, invasive pneumococcal disease; PCV, pneumococcal conjugate vaccine.

^a^PCV15 includes serotypes 1, 3, 4, 5, 6A/C, 6B, 7F, 9V, 14, 18C, 19F, 19A, 22F, 23F, 33F. PCV20 includes serotypes 1, 3, 4, 5, 6A/C, 6B, 7F, 8, 9V, 10A, 11A, 12F, 14, 15B, 18C, 19F, 19A, 22F, 23F, 33F. PCV21 includes serotypes 3, 6A/C, 7F, 8, 9N, 10A, 11A, 12F, 15A, 15C, 16F, 17F, 19A, 20A, 22F, 23A, 23B, 24F, 31, 33F, 35B. PCV24 includes serotypes 1, 2, 3, 4, 5, 6A/C, 6B, 7F, 8, 9N, 9V, 10A, 11A, 12F, 14, 15B, 17F, 18C, 19F, 19A, 20B, 22F, 23F, 33F. PCV25 includes serotypes 1, 2, 3, 4, 5, 6B, 6A/C, 7F, 8, 9N, 9V, 10A, 12F, 14, 15A, 15B, 16F, 18C, 19F, 19A, 22F, 23F, 24F, 33F, 35B. PCV31 includes serotypes 1, 2, 3, 4, 5, 6A/C, 6B, 7F, 7C, 8, 9N, 9V, 10A, 11A, 12F, 14, 15A, 15B, 16F, 17F, 18C, 19F, 19A, 20B, 22F, 23F, 23A, 23B, 31, 33F, 35B.

^b^Assuming no 15B and 15C cross-protection.

^c^Also includes Pn-MAPS24v.

^d^Sinusitis serotype distribution and PCV coverage estimates derived from meta-analysis of serotype distribution in pediatric acute otitis media and pneumonia.

^e^Nonbacteremic pneumonia. Bacteremic pneumonia included in IPD definition.

Pediatric coverage estimates increase markedly under the assumption that 15B-targeting vaccines (PCV20, PCV24, PCV25, PCV31) confer cross-protection against 15C (Supplementary Table 9) while adult estimates increased slightly (Supplementary Table 10). Serotype 15C accounts for 11.4% (95% CI, 10.5%–12.2%) of pediatric pneumococcal ARIs and 5.9% (95% CI, 4.6%–7.4%) of pediatric IPD, but <2% of adult pneumococcal pneumonia and approximately 1% of adult IPD (varying by age group; Figure 2, Supplementary Table 7).

### Potentially Preventable Burdens

Accounting for all-cause disease burdens (Supplementary Tables 11–13), serotype coverage (Supplementary Tables 14 and 15), and VE (Supplementary Table 6), PCV-preventable disease burdens among US children encompassed 58 189–784 825 outpatient-managed ARIs, 265–3547 nonbacteremic pneumonia hospitalizations, and 235–888 IPD cases annually (Table 3, Supplementary Table 16). The greatest potentially preventable burdens were associated with PCV31. When 15B/C cross-protection was considered, preventable burdens of pediatric pneumococcal ARIs increased notably while preventable pediatric IPD estimates increased slightly (Supplementary Table 17). Limited to children with only risk-based PCV indications, PCV21 could prevent 203 136 (95% CI, 138 227–293 061) outpatient-managed ARIs, 499 (95% CI, 296–757) nonbacteremic pneumonia hospitalizations, and 130 (95% CI, 103–157) IPD cases (Supplementary Table 8).

**Table 3.** Estimated Annual Number of Pneumococcal Disease Cases Potentially Preventable by Pneumococcal Conjugate Vaccines^a^ in the United States

Age Group and Condition	Annual No. of Cases in Thousands (95% CI)^b^					
	PCV15	PCV20	PCV21^c^	PCV24^d^	PCV25	PCV31
Children 0–17 y^e^						
AOM	47.3 (33.3–65.7)	213.8 (152.9–291.8)	…	246.9 (176.7–336.8)	354.0 (253.6–482.2)	639.4 (458.7–869.8)
Sinusitis^f^	7.1 (3.8–12.0)	31.9 (17.4–53.5)	…	36.9 (20.1–61.8)	52.9 (28.9–88.7)	95.1 (51.9–159.3)
Pneumonia (outpatient)^g^	3.4 (1.9–5.7)	15.4 (8.8–25.4)	…	17.9 (10.2–29.4)	25.6 (14.7–42.2)	46.1 (26.4–75.7)
Pneumonia (inpatient)^g^	0.3 (.2–.4)	1.2 (.7–1.8)	…	1.4 (.8–2.1)	2.0 (1.2–3.0)	3.5 (2.1–5.3)
IPD	0.2 (.2–.3)	0.4 (.4–.5)	…	0.5 (.4–.6)	0.6 (.5–.7)	0.9 (.8–1.0)
Adults ≥18 y^h^						
Sinusitis	162.7 (94.9–250.2)	731.2 (430.1–1108.6)	2675.0 (1576.4–4043.1)	845.6 (497.4–1281.4)	1214.0 (714.9–1837.6)	2181.8 (1285.7–3298.8)
Pneumonia (outpatient)^g^	46.1 (17.1–99.3)	109.7 (47–205.2)	263.9 (117.7–451.8)	179.9 (79.2–318.1)	156.9 (68.8–279.1)	307.9 (138–522.4)
Pneumonia (inpatient)^g^	2.0 (.7–4.3)	4.9 (2.1–9.1)	11.8 (5.3–20.0)	7.9 (3.5–13.9)	6.9 (3.1–12.3)	13.8 (6.2–23.1)
IPD	2.9 (1.8–3.6)	6.1 (3.7–7.6)	12.3 (7.5–15.2)	8.2 (5–10.2)	9.1 (5.6–11.3)	13.5 (8.2–16.7)

Abbreviations: AOM, acute otitis media; CI, confidence interval; IPD, invasive pneumococcal disease; PCV, pneumococcal conjugate vaccine.

^a^PCV15 includes serotypes 1, 3, 4, 5, 6A/C, 6B, 7F, 9V, 14, 18C, 19F, 19A, 22F, 23F, 33F. PCV20 includes serotypes 1, 3, 4, 5, 6A/C, 6B, 7F, 8, 9V, 10A, 11A, 12F, 14, 15B, 18C, 19F, 19A, 22F, 23F, 33F. PCV21 includes serotypes 3, 6A/C, 7F, 8, 9N, 10A, 11A, 12F, 15A, 15C, 16F, 17F, 19A, 20A, 22F, 23A, 23B, 24F, 31, 33F, 35B. PCV24 includes serotypes 1, 2, 3, 4, 5, 6A/C, 6B, 7F, 8, 9N, 9V, 10A, 11A, 12F, 14, 15B, 17F, 18C, 19F, 19A, 20B, 22F, 23F, 33F. PCV25 includes serotypes 1, 2, 3, 4, 5, 6B, 6A/C, 7F, 8, 9N, 9V, 10A, 12F, 14, 15A, 15B, 16F, 18C, 19F, 19A, 22F, 23F, 24F, 33F, 35B. PCV31 includes serotypes 1, 2, 3, 4, 5, 6A/C, 6B, 7F, 7C, 8, 9N, 9V, 10A, 11A, 12F, 14, 15A, 15B, 16F, 17F, 18C, 19F, 19A, 20B, 22F, 23F, 23A, 23B, 31, 33F, 35B.

^b^Considering United States population by age group [33] and assuming disease incidence rates as detailed in Supplementary Tables 11–13, pneumococcal-attributable disease proportions (Supplementary Table 4), preventable PCV coverage proportions of pneumococci by condition and age group (Supplementary Table 14), PCV effectiveness (Supplementary Table 6), and no serotype 15B and 15C cross-protection.

^c^Estimates for PCV21 for children 0–17 years not presented as PCV21 is not indicated for use in children.

^d^Also includes Pn-MAPS24v.

^e^Limited to children eligible for vaccination with PCV15 or PCV20: all children 0–4 years, and children 5–17 years at increased risk of pneumococcal disease (Supplementary Table 5). PCV15 or PCV20 currently recommended for all children 2–23 months, children 24–59 months with incomplete PCV vaccination status without contraindications for vaccination, and children 5–17 years with health conditions placing them at increased risk of pneumococcal disease [9, 10].

^f^Sinusitis only considered in children 5–17 years.

^g^Nonbacteremic pneumonia. Bacteremic pneumonia included in IPD definition.

^h^Limited to adults eligible for vaccination with PCV15, PCV20, or PCV21: all adults ≥50 years and adults 18–49 years at increased risk of pneumococcal disease (Supplementary Table 5). Vaccination with PCV15 (in addition to 23-valent pneumococcal polysaccharide vaccine [PPSV23]), PCV20, or PCV21 recommended for adults 18–49 years with health conditions placing them at increased risk of pneumococcal disease [11, 12]. Vaccination with PCV15 (plus PPSV23), PCV20, or PCV21 recommended for all adults ≥50 years without contraindications for vaccination [11, 12].

In US PCV-eligible adults, potentially preventable disease burdens included 211 888–2 496 235 outpatient-managed ARIs, 1979–13 751 nonbacteremic pneumonia hospitalizations, and 2913–13 464 IPD cases annually (Table 3, Supplementary Table 18). The greatest preventable burdens of adult nonbacteremic pneumonia and IPD were from PCV31. Limited increases in the estimated preventable burden of adult pneumonia and IPD were observed with 15B/C cross-protection (Supplementary Table 19).

In total, we estimated that existing and pipeline PCV products could prevent 270 473–3 284 917 outpatient ARI visits, 2249–17 348 pneumonia hospitalizations, and 2998–13 607 IPD cases, relative to the burden of disease observed across all age groups in 2019, under use cases aligned with current age- and risk-based recommendations (Table 4). In sensitivity analyses varying estimated VE, a 10% reduction in VE resulted in preventable burdens of 243 426–2 956 426 outpatient ARI visits, 2024–15 614 pneumonia hospitalizations, and 2698–12 247 IPD cases while a 10% increase in VE yielded preventable burdens of 297 521–3 613 409 outpatient ARI visits, 2474–19 083 pneumonia hospitalizations, and 3298–14 968 IPD cases. When assumed VE was decreased by 25%, potentially preventable burdens further decreased to 202 855–2 463 688 outpatient ARI visits, 1687–13 011 pneumonia hospitalizations, and 2249–10 206 IPD cases. Conversely, increasing VE by 25% yielded potentially preventable burdens of 338 092–4 106 147 outpatient ARI visits, 2811–21 685 pneumonia hospitalizations, and 3748–17 009 IPD cases.

**Table 4.** Sensitivity Analysis of Estimated Annual Number of Pneumococcal Disease Cases Potentially Preventable by Pneumococcal Conjugate Vaccines^a^ in the United States With Varying Vaccine Effectiveness Estimates

Condition^a^	Pneumococcal Vaccine^b^	Annual No. of Cases in Thousands (95% CI) by VE Scenario				
		Base Case	10% Lower	25% Lower	10% Higher	25% Higher
AOM^c^	PCV15	47.3 (33.3–65.7)	42.6 (30.0–59.2)	35.5 (25–49.3)	52.1 (36.7–72.3)	59.2 (41.7–82.2)
	PCV20	213.8 (152.9–291.8)	192.4 (137.6–262.6)	160.3 (114.7–218.8)	235.1 (168.2–320.9)	267.2 (191.1–364.7)
	PCV21^d^	…	…	…	…	…
	PCV24	246.9 (176.7–336.8)	222.2 (159.0–303.1)	185.2 (132.5–252.6)	271.6 (194.4–370.5)	308.7 (220.9–421.0)
	PCV25	354.0 (253.6–482.2)	318.6 (228.3–434.0)	265.5 (190.2–361.7)	389.3 (279.0–530.4)	442.4 (317.0–602.8)
	PCV31	639.4 (458.7–869.8)	575.5 (412.8–782.8)	479.6 (344.0–652.3)	703.4 (504.6–956.7)	799.3 (573.4–1087.2)
Sinusitis^e^	PCV15	170.0 (99.2–261.0)	153.0 (89.3–234.9)	127.5 (74.4–195.8)	187.0 (109.1–287.1)	212.5 (124–326.3)
	PCV20	763.8 (449.6–1156.3)	687.4 (404.6–1040.7)	572.8 (337.2–867.2)	840.1 (494.5–1271.9)	954.7 (562.0–1445.4)
	PCV21^d^	2675.0 (1576.4–4043.1)	2407.5 (1418.8–3638.8)	2006.2 (1182.3–3032.3)	2942.5 (1734.0–4447.4)	3343.7 (1970.5–5053.8)
	PCV24	883.2 (520–1336.5)	794.9 (468.0–1202.9)	662.4 (390.0–1002.4)	971.5 (572.0–1470.2)	1104.0 (650.0–1670.7)
	PCV25	1268 (747.3–1916.4)	1141.2 (672.6–1724.8)	951.0 (560.5–1437.3)	1394.8 (822.1–2108.1)	1585.0 (934.2–2395.6)
	PCV31	2278.7 (1343.8–3440.1)	2050.9 (1209.4–3096.1	1709.1 (1007.9–2580.1)	2506.6 (1478.2–3784.1)	2848.4 (1679.8–4300.1)
Pneumonia (outpatient)^f^	PCV15	49.7 (20.6–102.9)	44.7 (18.5–92.6)	37.2 (15.4–77.2)	54.6 (22.6–113.2)	62.1 (25.7–128.6)
	PCV20	125.6 (62.3–221.4)	113.1 (56.1–199.2)	94.2 (46.7–166.0)	138.2 (68.5–243.5)	157.0 (77.9–276.7)
	PCV21^d^	263.9 (117.7–451.8)	237.5 (105.9–406.7)	197.9 (88.3–338.9)	290.3 (129.5–497.0)	329.9 (147.1–564.8)
	PCV24	198.2 (97.0–336.8)	178.4 (87.3–303.1)	148.7 (72.8–252.6)	218.1 (106.7–370.5)	247.8 (121.3–421.0)
	PCV25	183.3 (94.1–306.2)	165.0 (84.6–275.6)	137.5 (70.5–229.7)	201.7 (103.5–336.8)	229.2 (117.6–382.8)
	PCV31	355.3 (183.4–571.2)	319.8 (165.1–514.1)	266.5 (137.6–428.4)	390.8 (201.8–628.4)	444.1 (229.3–714.0)
All outpatient^g^	PCV15	270.5 (184.3–378.7)	243.4 (165.8–340.8)	202.9 (138.2–284.0)	297.5 (202.7–416.5)	338.1 (230.3–473.3)
	PCV20	1109.2 (761.3–1537.6)	998.3 (685.2–1383.9)	831.9 (571.0–1153.2)	1220.1 (837.4–1691.4)	1386.5 (951.6–1922.0)
	PCV21^d^	2944.8 (1832.0–4322.4)	2650.3 (1648.8–3890.2	2208.6 (1374.0–3241.8)	3239.3 (2015.2–4754.7)	3681.0 (2290.0–5403.1)
	PCV24	1335.6 (925.9–1835.9)	1202.0 (833.3–1652.3)	1001.7 (694.5–1376.9)	1469.1 (1018.5–2019.5)	1669.5 (1157.4–2294.9)
	PCV25	1812.8 (1242.0–2514.6)	1631.5 (1117.8–2263.1)	1359.6 (931.5–1885.9)	1994.1 (1366.2–2766.0)	2266.0 (1552.5–3143.2)
	PCV31	3284.9 (2259.0–4541.9)	2956.4 (2033.1–4087.7)	2463.7 (1694.3–3406.4)	3613.4 (2484.9–4996.1)	4106.1 (2823.8–5677.4)
Pneumonia (inpatient)^f^	PCV15	2.2 (1.0–4.5)	2.0 (.9–4.1)	1.7 (.7–3.4)	2.5 (1.1–5.0)	2.8 (1.2–5.7)
	PCV20	6.1 (3.3–10.4)	5.5 (2.9–9.3)	4.6 (2.4–7.8)	6.7 (3.6–11.4)	7.7 (4.1–13.0)
	PCV21^d^	11.8 (5.3–20.0)	10.6 (4.7–18.0)	8.8 (4.0–15.0)	12.9 (5.8–22.0)	14.7 (6.6–25.0)
	PCV24	9.3 (4.9–15.4)	8.4 (4.4–13.8)	7.0 (3.6–11.5)	10.3 (5.3–16.9)	11.7 (6.1–19.2)
	PCV25	8.9 (4.9–14.3)	8.1 (4.5–12.9)	6.7 (3.7–10.8)	9.8 (5.4–15.8)	11.2 (6.2–17.9)
	PCV31	17.3 (9.6–26.8)	15.6 (8.6–24.2)	13.0 (7.2–20.1)	19.1 (10.6–29.5)	21.7 (12.0–33.6)
IPD	PCV15	3.0 (1.9–3.7)	2.7 (1.7–3.3)	2.2 (1.4–2.8)	3.3 (2.1–4.1)	3.7 (2.4–4.6)
	PCV20	6.2 (3.9–7.5)	5.5 (3.5–6.8)	4.6 (2.9–5.7)	6.8 (4.3–8.3)	7.7 (4.9–9.4)
	PCV21^d^	11.6 (7.1–14.3)	10.4 (6.4–12.9)	8.7 (5.3–10.8)	12.7 (7.8–15.8)	14.5 (8.8–17.9)
	PCV24	8.2 (5.2–10.0)	7.4 (4.7–9.0)	6.1 (3.9–7.5)	9.0 (5.7–11.0)	10.2 (6.5–12.5)
	PCV25	9.3 (5.9–11.3)	8.3 (5.3–10.2)	6.9 (4.4–8.5)	10.2 (6.5–12.5)	11.6 (7.4–14.2)
	PCV31	13.6 (8.6–16.6)	12.2 (7.8–15.0)	10.2 (6.5–12.5)	15.0 (9.5–18.3)	17.0 (10.8–20.8)

Abbreviations: AOM, acute otitis media; CI, confidence interval; IPD, invasive pneumococcal disease; PCV, pneumococcal conjugate vaccine; VE, vaccine effectiveness.

^a^All ages unless otherwise indicated. Limited to children eligible for vaccination with PCV15 or PCV20 (all children 0–4 years, and children 5–17 years at increased risk of pneumococcal disease) and adults eligible for vaccination with PCV15, PCV20, or PCV21 (all adults ≥50 years and adults 18–49 years at increased risk of pneumococcal disease; Supplementary Table 5). PCV15 or PCV20 currently recommended for all children 2–23 months, children 24–59 months with incomplete PCV vaccination status without contraindications for vaccination, and children 5–17 years with health conditions placing them at increased risk of pneumococcal disease [9, 10]. Vaccination with PCV15 (plus 23-valent pneumococcal polysaccharide vaccine [PPSV23], PCV20, or PCV21 recommended for adults 18–49 years with health conditions placing them at increased risk of pneumococcal disease [11, 12]. Vaccination with PCV15 (plus PPSV23), PCV20, or PCV21 recommended for all adults ≥50 years without contraindications for vaccination [11, 12].

^b^PCV15 includes serotypes 1, 3, 4, 5, 6A/C, 6B, 7F, 9V, 14, 18C, 19F, 19A, 22F, 23F, 33F. PCV20 includes serotypes 1, 3, 4, 5, 6A/C, 6B, 7F, 8, 9V, 10A, 11A, 12F, 14, 15B, 18C, 19F, 19A, 22F, 23F, 33F. PCV21 includes serotypes 3, 6A/C, 7F, 8, 9N, 10A, 11A, 12F, 15A, 15C, 16F, 17F, 19A, 20A, 22F, 23A, 23B, 24F, 31, 33F, 35B. PCV24 includes serotypes 1, 2, 3, 4, 5, 6A/C, 6B, 7F, 8, 9N, 9V, 10A, 11A, 12F, 14, 15B, 17F, 18C, 19F, 19A, 20B, 22F, 23F, 33F. PCV25 includes serotypes 1, 2, 3, 4, 5, 6B, 6A/C, 7F, 8, 9N, 9V, 10A, 12F, 14, 15A, 15B, 16F, 18C, 19F, 19A, 22F, 23F, 24F, 33F, 35B. PCV31 includes serotypes 1, 2, 3, 4, 5, 6A/C, 6B, 7F, 7C, 8, 9N, 9V, 10A, 11A, 12F, 14, 15A, 15B, 16F, 17F, 18C, 19F, 19A, 20B, 22F, 23F, 23A, 23B, 31, 33F, 35B.

^c^Estimates for AOM only include children aged 0–17 years eligible for PCV vaccination.

^d^Estimates for PCV21 for children aged 0–17 years not presented as PCV21 is not indicated for use in children.

^e^Estimates for sinusitis include only children aged 5–17 years and adults aged ≥18 years eligible for PCV vaccination.

^f^Nonbacteremic pneumonia. Bacteremic pneumonia included in IPD definition.

^g^Includes AOM, sinusitis, and outpatient-managed nonbacteremic pneumonia.

## DISCUSSION

For next-generation PCVs, we found that serotype coverage and preventable burdens varied widely by condition and age group. Overall, pneumococcal disease coverage was lowest for PCV15 and highest for PCV31. PCV20 offered nearly 2-fold greater coverage of pediatric ARIs compared with PCV15; differences for IPD and adult nonbacteremic pneumonia were modest. In pediatric ARIs, PCV24 offered minimal coverage improvements over PCV20; larger increases were observed with PCV25. In adult disease, coverage from PCV24 and PCV25 were similar. Notably, PCV21 provided greater coverage than all other pipeline vaccines except PCV31 for adult pneumonia and IPD. Across PCVs, preventable burdens encompassed 270 000 to 3.3 million outpatient-managed ARIs, 2000–17 000 nonbacteremic pneumonia hospitalizations, and 3000–14 000 IPD cases in the United States each year.

We found that the most prevalent serotypes in pediatric pneumococcal ARIs are 15C, 23B, 11A, 15A, and 35B. No PCVs except PCV21, which is not currently indicated for pediatric use, target all of these serotypes. However, PCV31 targets 23B, 11A, 15A, 35B, and 15B, with potential 15C cross-reactivity. In adult sinusitis (where serotype distribution was inferred from pediatric ARIs), PCV21 provides greater coverage than PCV31 from inclusion of 15C. The most prevalent model-estimated serotypes in adult nonbacteremic pneumonia are 3, 22F, 20B, 19A, and 35B. However, in adults ≥65 years, universally eligible for PCV immunization [11], 20B accounts for only 3% of pneumococcal pneumonia. All PCVs target 22F and PCV21, PCV25, PCV31 target 35B while only PCV31 targets 20B. Serotypes 22Fand 33F, common in pediatric IPD, are included in all PCV formulations while 23B, also common in pediatric IPD is only targeted by PCV31 and PCV21 (not indicated for children). Serotypes 3 and 22F are also top contributors to adult IPD, along with 23A, 9N, and 8. PCV21 and PCV31 target 23A and PCV21, PCV24, PCV25, and PCV31 target 9N and 8.

Increases in valency have corresponded to numerically lower serotype-specific immune responses, a phenomenon known as carrier suppression. Thus, serotype coverage added to existing conjugate protein delivery systems may be offset by diminishing immune response. In a phase 3 trial of PCV20, immune responses 1 month after the third priming dose failed to meet noninferiority criteria for 5 serotypes common to PCV13 and PCV20 [39]. In a phase 3 trial among adults, PCV20-elicited opsonophagocytic activity (OPA) geometric mean fold rises met noninferiority criteria, but were numerically lower than those from PCV13 for most (11/13) shared serotypes [40]. However, it is unknown if immune response differences translate into VE differences. Whether new protein conjugation methods (PCV24, PCV31) and MAPS technology (PN-MAPS24v) mitigate carrier suppression remains to be determined. In dose-ranging studies, immunogenicity of PCV24 delivering 2.2 µg of each antigen was equal to or greater than PCV20-induced immunogenicity for shared serotypes, although carrier suppression occurred in a product delivering 4.4 µg for 7 antigens [41, 42]. Data from phase 1 and 2 trials suggest robust immune responses to Pn-MAPS24v [43, 44]. Although employing a traditional platform, PCV21 mitigates carrier suppression by dropping select serotypes targeted by existing PCVs. In estimating preventable burdens, we assume syndrome-specific VEs equivalent to PCV7/PCV13; the effects of carrier suppression on this assumption remain to be determined. Postlicensure VE studies are needed to evaluate whether differences in immunogenicity will translate into differences in VE against IPD and ARIs among the PCVs considered here. In sensitivity analyses, we found that 10% and 25% variations in VE, in line with previously estimated ranges of differences in VE point estimates against carriage between PCV7 and PCV13 for shared serotypes [45], were associated with marked increases and reductions in potentially preventable burdens.

Serotype cross-protection is also an important unknown. Better understanding of 15B/C cross-protection is needed to evaluate potential PCV impacts on pediatric ARIs, where 15C is prevalent. While a study of children aged 6–36 months found no 15B/C functional antibody cross-reactivity associated with prior colonization [46], elevated OPA titers against 15C were identified among adults aged 18–49 years immunized with PCV20 [26]. Additionally, a phase 3 trial demonstrated OPA responses to 15B among adults immunized with PCV21 [47]. Notably, PCV21 is indicated for prevention of serotype 15B and 15C IPD, but only for serotype 15B pneumonia [20], for which protection requires higher antibody levels. We assumed complete 6A/C cross-protection based on demonstrated PCV13 cross-protection in AOM and IPD [24].

Our analysis has limitations. First, we use studies from US and non-US contexts to estimate pediatric ARI serotype distribution. Inclusion of non-US studies from comparable contexts allowed for robust estimation. However, potential geographic variation in serotype distribution could affect our estimates.

Second, we relied on AOM studies to inform serotype distribution estimates for sinusitis and pediatric nonbacteremic pneumonia given limited available data for these conditions. Similarly, we extrapolated PCV VE against sinusitis from AOM. Third, we estimate serotype distribution in AOM from studies using nasopharyngeal sampling. Optimal frameworks for etiologic determinations in ARI are lacking; nasopharyngeal samples may capture commensals in addition to causative agents, whereas middle ear fluid (MEF) sampling may be biased toward children with recurrent or complex AOM. A study conducted in children with AOM sampling both from the nasopharynx and MEF found slight differences in serotype distribution between methods [48]. Additionally, in previous work we demonstrated higher proportions of PCV13 serotypes in studies of MEF compared with nasopharyngeal samples [30]. Fourth, adult nonbacteremic pneumonia serotype distribution was estimated from adults hospitalized in 2 major health systems in the southeastern United States and may not be nationally representative or translate to outpatient-managed pneumonia. Geographic variation is important for serotype 4, for which IPD incidence increased in 3 of 10 regional ABCs sites from 2010 to 2018 [49] and which is in all PCVs except PCV21. Fifth, serotypes causing IPD informed estimated adult nonbacteremic pneumonia serotype distribution: The PNEUMO study included bacteremic pneumococcal pneumonia cases (14.5%) and we inferred non-SSUAD serotype distribution using IPD data. Sixth, base-case burden estimates rely on overall PCV7/PCV13 VE and do not consider serotype-specific VE, future serotype replacement, or indirect effects from pediatric immunization. We assume equivalent PCV13-serotype VE for all PCVs and exclude PCV13 serotypes from preventable burden estimates, potentially underestimating preventable burdens. Underestimation may be consequential for serotype 3, which accounts for sizeable proportions of adult disease and for which PCV13 VE estimates remain imprecise [22]. Finally, post-COVID-19 pandemic healthcare utilization for pneumococcal diseases is unknown. Despite these limitations, our study provides a comprehensive analysis of best-available data to estimate the burden of pneumococcal disease in the US preventable by PCV products across multiple conditions.

In summary, we observed wide variation across PCV products in serotype coverage and potentially preventable burdens. PCV21, PCV24, PCV25, and PCV31 target serotypes accounting for >60% of IPD and adult pneumonia with lesser coverage of pediatric ARIs. Potentially preventable burdens were lowest for PCV15 and highest for PCV31 across all syndromes and age groups. Among adults, PCV21 also prevented notable burdens of both ARIs and IPD. Variation in both invasive and mucosal disease coverage and preventable burdens may be an important consideration in product recommendations as a wider variety of next-generation PCV formulations becomes available.

## Supplementary Material

jiaf376_Supplementary_Data

## Supplementary Data

Supplementary materials are available at *The Journal of Infectious Diseases* online (http://jid.oxfordjournals.org/). Supplementary materials consist of data provided by the author that are published to benefit the reader. The posted materials are not copyedited. The contents of all supplementary data are the sole responsibility of the authors. Questions or messages regarding errors should be addressed to the author.

## Notes

***Author contributions.*** L. M. K.: Conceptualization, methodology, validation, formal analysis, investigation, data curation, writing—original draft, visualization. K. L. A.: Conceptualization, resources, writing—review & editing, supervision, project administration. M. K. and A. L. C.: Conceptualization, resources, writing—review & editing. W. X.: Formal analysis, writing—review & editing. W. H. S., J. J. R., C. G. W., A. B., M. K., C. G. G., J. T., and N. R.: Investigation, writing—review & editing. J. A. L.: Conceptualization, methodology, writing—review & editing, supervision, resources, funding acquisition.

***Acknowledgments.*** The authors would like to thank Dr Jin Han, Dr Kelly Johnson, Dr Thomas Weiss, Dr Craig Roberts, and all other PNEUMO Study Investigators for their contributions to the PNEUMO study, which contributed data for this analysis.

***Disclaimer.*** The National Institutes of Health (NIH) had no input into the design and conduct of the study; collection, management, analysis, and interpretation of the data; preparation, review, or approval of the manuscript; and decision to submit the manuscript for publication. The Centers of Disease Control and Prevention (CDC) was involved through co-author participation the design and conduct of the study; analysis and interpretation of the data; preparation, review, or approval of the manuscript; and decision to submit the manuscript for publication. The content is solely the responsibility of the authors and does not necessarily represent the official views of the NIH and the CDC.

***Financial support.*** This work was supported by the Centers for Disease Control and Prevention (grant number 21IPA2111845 to J. A. L.) and the National Institutes of Health (grant number 1F31AI174773 to L. M. K.).

## References

[1] Blacklock CB, Weinberger DM, Perniciaro S, Wyllie AL. *Streptococcus pneumoniae* serotypes. **2024**. Available at: https://pneumococcalcapsules.github.io/serotypes/. Accessed 1 May 2025.

[2] Varghese J, Chochua S, Tran T, et al Multistate population and whole genome sequence-based strain surveillance of invasive pneumococci recovered in the USA during 2017. Clin Microbiol Infect 2020; 26:512.e1–e10. 10.1016/j.cmi.2019.09.008 PMC12019393 31536818

[3] Centers for Disease Control and Prevention . Bact facts: *Streptococcus pneumoniae*. Available at: https://app.powerbigov.us/view?r=eyJrIjoiNjc5OGRjODctNWQ5ZC00ZWEwLWI5ZjgtNGI3ZmFhODVmYTlhIiwidCI6IjljZTcwODY5LTYwZGItNDRmZC1hYmU4LWQyNzY3MDc3ZmM4ZiJ9&pageName=ReportSectione93482d78e7dc3ed111b. Accessed 8 March 2022.

[4] Hersh AL, King LM, Shapiro DJ, Hicks LA, Fleming-Dutra KE. Unnecessary antibiotic prescribing in US ambulatory care settings, 2010–2015. Clin Infect Dis 2021; 72:133–7. 32484505 10.1093/cid/ciaa667 PMC9377284

[5] King LM, Tsay SV, Hicks LA, Bizune D, Hersh AL, Fleming-Dutra K. Changes in outpatient antibiotic prescribing for acute respiratory illnesses, 2011 to 2018. Antimicrob Steward Healthc Epidemiol 2021; 1:1–8. 35923647 10.1017/ash.2021.230 PMC9345578

[6] Eskola J, Kilpi T, Palmu A, et al Efficacy of a pneumococcal conjugate vaccine against acute otitis media. N Engl J Med 2001; 344:403–9. 11172176 10.1056/NEJM200102083440602

[7] Lewnard JA, Givon-Lavi N, Dagan R. Dose-specific effectiveness of 7- and 13-valent pneumococcal conjugate vaccines against vaccine-serotype *Streptococcus pneumoniae* colonization in children. Clin Infect Dis 2020; 71:e289–300. 31784753 10.1093/cid/ciz1164 PMC8463090

[8] Lewnard JA, Bruxvoort KJ, Hong VX, et al Effectiveness of pneumococcal conjugate vaccination against virus-associated lower respiratory tract infection among adults: a case-control study. J Infect Dis 2023:227:498–511. 35323906 10.1093/infdis/jiac098 PMC9383607

[9] ACIP updates: recommendations for use of 20-valent pneumococcal conjugate vaccine in children—United States, 2023. MMWR Morb Mortal Wkly Rep 2023;72:1072. 37768876 10.15585/mmwr.mm7239a5 PMC10545431

[10] Kobayashi M, Farrar JL, Gierke R, et al Use of 15-valent pneumococcal conjugate vaccine among U.S. children: updated recommendations of the Advisory Committee on Immunization Practices—United States, 2022. MMWR Morb Mortal Wkly Rep 2022; 71:1174–81. 36107786 10.15585/mmwr.mm7137a3 PMC9484809

[11] Kobayashi M, Pilishvili T, Farrar JL, et al Pneumococcal vaccine for adults aged ≥19 years: recommendations of the Advisory Committee on Immunization Practices, United States, 2023. MMWR Recomm Rep 2023; 72:1–39. 10.15585/mmwr.rr7203a1 PMC10495181 37669242

[12] Centers for Disease Control and Prevention . ACIP recommendations. Advisory Committee on Immunization Practices (ACIP). **2024**. Available at: https://www.cdc.gov/acip/vaccine-recommendations/index.html. Accessed 25 October 2024.

[13] Kandasamy R, Voysey M, Collins S, et al Persistent circulation of vaccine serotypes and serotype replacement after 5 years of infant immunization with 13-valent pneumococcal conjugate vaccine in the United Kingdom. J Infect Dis 2020; 221:1361–70. 31004136 10.1093/infdis/jiz178

[14] Weinberger DM, Malley R, Lipsitch M. Serotype replacement in disease after pneumococcal vaccination. Lancet 2011; 378:1962–73. 21492929 10.1016/S0140-6736(10)62225-8 PMC3256741

[15] Lewnard JA, Hanage WP. Making sense of differences in pneumococcal serotype replacement. Lancet Infect Dis 2019; 19:e213–20. 30709666 10.1016/S1473-3099(18)30660-1

[16] Lewnard JA, Givon-Lavi N, Dagan R. Effectiveness of pneumococcal conjugate vaccines against community-acquired alveolar pneumonia attributable to vaccine-serotype *Streptococcus pneumoniae* among children. Clin Infect Dis 2021; 73:e1423–33. 33346348 10.1093/cid/ciaa1860 PMC8492210

[17] Pimenta F, Moiane B, Gertz RE, et al New pneumococcal serotype 15D. J Clin Microbiol 2021; 59:e00329–1. 33658265 10.1128/JCM.00329-21 PMC8091843

[18] Centers for Disease Control and Prevention . Active Bacterial Core surveillance (ABCs). 2**024**. Available at: https://www.cdc.gov/abcs/about/index.html. Accessed 18 September 2024.

[19] Self WH, Johnson KD, Resser JJ, et al Prevalence, clinical severity, and serotype distribution of pneumococcal pneumonia among adults hospitalized with community-acquired pneumonia in Tennessee and Georgia, 2018–2022. Clin Infect Dis 2024; 79:838–47. 39016606 10.1093/cid/ciae316 PMC11478805

[20] US Food and Drug Administration . CAPVAXIVE. 2**024**. Available at: https://www.fda.gov/vaccines-blood-biologics/capvaxive. Accessed 17 October 2024.

[21] Merck Sharp & Dohme LLC . A phase 3, randomized, double-blind study to evaluate the safety, tolerability, and immunogenicity of V116 in children and adolescents with increased risk of pneumococcal disease. 2024. Available at: https://clinicaltrials.gov/study/NCT06177912. Accessed 17 October 2024. 10.1080/21645515.2026.2684089 PMC13313238 42334402

[22] Andrejko KL, Gierke R, Rowlands JV, et al Effectiveness of 13-valent pneumococcal conjugate vaccine for prevention of invasive pneumococcal disease among children in the United States between 2010 and 2019: an indirect cohort study. Vaccine 2024; 42:3555–63. 38704263 10.1016/j.vaccine.2024.04.061 PMC11288330

[23] Cooper D, Yu X, Sidhu M, Nahm MH, Fernsten P, Jansen KU. The 13-valent pneumococcal conjugate vaccine (PCV13) elicits cross-functional opsonophagocytic killing responses in humans to *Streptococcus pneumoniae* serotypes 6C and 7A. Vaccine 2011; 29:7207–11. 21689707 10.1016/j.vaccine.2011.06.056 PMC3170457

[24] Grant LR, Hanquet G, Sepúlveda-Pachón IT, et al Effects of PCV10 and PCV13 on pneumococcal serotype 6C disease, carriage, and antimicrobial resistance. Vaccine 2024; 42:2983–93. 38553292 10.1016/j.vaccine.2024.03.065

[25] Self WH, Rouphael N, Resser JJ, Johnson KD. Interim results from the PNEUMO Study. In: Advisory Committee on Immunization Practices, 29 February 2024, Atlanta, Georgia, USA. Available at: https://www.cdc.gov/vaccines/acip/meetings/downloads/slides-2024-02-28-29/03-Pneumococcal-Self-508.pdf. Accessed 2 April 2024.

[26] Hao L, Kuttel MM, Ravenscroft N, et al *Streptococcus pneumoniae* serotype 15B polysaccharide conjugate elicits a cross-functional immune response against serotype 15C but not 15A. Vaccine 2022; 40:4872–80. 35810060 10.1016/j.vaccine.2022.06.041

[27] Tamimi N, Kline MJ, Center KJ, et al Immune responses to cross-reactive serotypes 6C and 15C after 20-valent pneumococcal conjugate vaccine in infants. Open Forum Infect Dis 2023; 10(Suppl 2):ofad500.1560.

[28] Centers for Disease Control and Prevention . Active Bacterial Core surveillance (ABCs) Report. Emerging Infections Program Network: *Streptococcus pneumoniae*, 2019. Available at: https://www.cdc.gov/abcs/downloads/spn_surveillance_report_2019.pdf. Accessed 27 June 2024.

[29] Healthcare Cost and Utilization Project. Overview of the National (Nationwide) Inpatient Sample (NIS) . 2025. Available at: https://hcup-us.ahrq.gov/nisoverview.jsp. Accessed 26 June 2024.

[30] King LM, Andrejko KL, Kabbani S, et al Outpatient visits and antibiotic use due to higher-valency pneumococcal vaccine serotypes. J Infect Dis 2024; 230:821–31. 38498565 10.1093/infdis/jiae142 PMC11481348

[31] Lewnard JA, King LM, Fleming-Dutra KE, Link-Gelles R, Van Beneden CA. Incidence of pharyngitis, sinusitis, acute otitis media, and outpatient antibiotic prescribing preventable by vaccination against group A *Streptococcus* in the United States. Clin Infect Dis 2021; 73:e47–58. 32374829 10.1093/cid/ciaa529

[32] Centers for Disease Control and Prevention . 2019 NAMCS micro-data file documentation. **2019**. Accessed 26 June 2024. Available at: https://ftp.cdc.gov/pub/Health_Statistics/NCHS/Dataset_Documentation/NAMCS/doc2019-508.pdf.

[33] Centers for Disease Control and Prevention . Bridged-race population estimates—data files and documentation. 2**021**. Available at: https://www.cdc.gov/nchs/nvss/bridged_race/data_documentation.htm. Accessed 12 June 2023.

[34] Centers for Disease Control and Prevention . ACIP vaccine recommendations and schedules. 2**024**. Available at: https://www.cdc.gov/vaccines/acip/recommendations.html. Accessed 11 September 2024.

[35] Lucero MG, Dulalia VE, Parreño RAN, et al Pneumococcal conjugate vaccines for preventing vaccine-type invasive pneumococcal disease and pneumonia with consolidation on x-ray in children under two years of age. Available at: https://www.cochranelibrary.com/cdsr/doi/10.1002/14651858.CD004977/full. Accessed 18 September 2024. 10.1002/14651858.CD004977 15495133

[36] Klugman KP, Rodgers GL. Impact of pneumococcal conjugate vaccine on vaccine serotype–specific pneumonia. Clin Infect Dis 2021; 73:e1434–5. 33338195 10.1093/cid/ciaa1867 PMC8492200

[37] Prasad N, Stoecker C, Xing W, Cho BH, Leidner AJ, Kobayashi M. Public health impact and cost-effectiveness of 15-valent pneumococcal conjugate vaccine use among the pediatric population of the United States. Vaccine 2023; 41:2914–21. 37012118 10.1016/j.vaccine.2023.03.045 PMC10962013

[38] Bonten MJM, Huijts SM, Bolkenbaas M, et al Polysaccharide conjugate vaccine against pneumococcal pneumonia in adults. N Engl J Med 2015; 372:1114–25. 25785969 10.1056/NEJMoa1408544

[39] Senders S, Klein NP, Tamimi N, et al A phase three study of the safety and immunogenicity of a four-dose series of 20-valent pneumococcal conjugate vaccine in healthy infants. Pediatr Infect Dis J 2024; 43:596–603. 38535409 10.1097/INF.0000000000004334 PMC11090512

[40] Essink B, Sabharwal C, Cannon K, et al Pivotal phase 3 randomized clinical trial of the safety, tolerability, and immunogenicity of 20-valent pneumococcal conjugate vaccine in adults aged ≥18 years. Clin Infect Dis 2022; 75:390–8. 34940806 10.1093/cid/ciab990 PMC9427137

[41] Wassil J, Sisti M, Fairman J, et al Evaluating the safety, tolerability, and immunogenicity of a 24-valent pneumococcal conjugate vaccine (VAX-24) in healthy adults aged 18 to 64 years: a phase 1/2, double-masked, dose-finding, active-controlled, randomised clinical trial. Lancet Infect Dis 2023; 24:308–18. 38061367 10.1016/S1473-3099(23)00572-8

[42] Wassil J, Sisti M, Fairman J, et al A phase 2, randomized, blinded, dose-finding, controlled clinical trial to evaluate the safety, tolerability, and immunogenicity of a 24-valent pneumococcal conjugate vaccine (VAX-24) in healthy adults 65 years and older. Vaccine 2024; 42:126124. 39025698 10.1016/j.vaccine.2024.07.025

[43] Chichili GR, Smulders R, Santos V, et al Phase 1/2 study of a novel 24-valent pneumococcal vaccine in healthy adults aged 18 to 64 years and in older adults aged 65 to 85 years. Vaccine 2022; 40:4190–8. 35690500 10.1016/j.vaccine.2022.05.079

[44] Borys D, Rupp R, Smulders R, et al Safety, tolerability and immunogenicity of a novel 24-valent pneumococcal vaccine in toddlers: a phase 1 randomized controlled trial. Vaccine 2024; 42:2560–71. 38360475 10.1016/j.vaccine.2024.02.001

[45] Dagan R, Patterson S, Juergens C, et al Comparative immunogenicity and efficacy of 13-valent and 7-valent pneumococcal conjugate vaccines in reducing nasopharyngeal colonization: a randomized double-blind trial. Clin Infect Dis 2013; 57:952–62. 23804191 10.1093/cid/cit428

[46] Kaur R, Gonzalez E, Pham M, Pichichero M. Naturally-induced serum antibody levels in children to pneumococcal polysaccharide 15B that correlate with protection from nasopharyngeal colonization but anti-serotype 15B antibody has low functional cross-reactivity with serotype 15C. Vaccine 2023; 41:7265–73. 37925318 10.1016/j.vaccine.2023.10.054

[47] Platt HL, Bruno C, Buntinx E, et al Safety, tolerability, and immunogenicity of an adult pneumococcal conjugate vaccine, V116 (STRIDE-3): a randomised, double-blind, active comparator controlled, international phase 3 trial. Lancet Infect Dis 2024; 24:1141–50. 38964361 10.1016/S1473-3099(24)00344-X

[48] Kaur R, Schulz S, Sherman A, Andrejko K, Kobayashi M, Pichichero M. Anticipated effects of higher-valency pneumococcal conjugate vaccines on colonization and acute otitis media. Pediatr Infect Dis J 2024; 43:1004–10. 38838209 10.1097/INF.0000000000004413 PMC11408086

[49] Beall B, Walker H, Tran T, et al Upsurge of conjugate vaccine serotype 4 invasive pneumococcal disease clusters among adults experiencing homelessness in California, Colorado, and New Mexico. J Infect Dis 2021; 223:1241–9. 32798216 10.1093/infdis/jiaa501 PMC8108119

