# Supplementary material for: Pneumococcal Serotype Distribution and Coverage of Existing and Pipeline Pneumococcal Vaccines
Source: J Infect Dis. 2025 Jul 22;232(4):e609–20. doi: 10.1093/infdis/jiaf376 (PMC12526911; doi:10.1093/infdis/jiaf376)
Supplement: jiaf376_Supplementary_Data [file jiaf376_supplementary_data.pdf]

SUPPLEMENTAL MATERIAL

Pneumococcal serotype distribution and coverage of existing and pipeline pneumococcal vaccines

Laura M King,[1] Kristin L Andrejko,[2] Miwako Kobayashi,[2] Wei Xing,[2] Adam L Cohen,[2] Wesley H Self,[3] J Jackson Resser,[3] Cynthia G Whitney,[4] Adrienne Baughman,[3] Mai Kio,[5] Carlos G Grijalva,[3] Jessica Traenkner,[5] Nadine Rouphael,[5] Joseph A Lewnard[1]

[1] School of Public Health, University of California, Berkeley, Berkeley, California, United States; [2] Division of Bacterial Diseases, Centers for Disease Control and Prevention, Atlanta, Georgia, United States; [3] Vanderbilt University Medical Center, Nashville, Tennessee, United States; [4] Rollins School of Public Health, Emory University, Atlanta, Georgia, United States; [5] Department of Medicine, Emory University, Atlanta, Georgia, United States

Corresponding author:
Laura King
University of California, Berkeley
2121 Berkeley Way
Berkeley, CA 94704
laura_king@berkeley.edu

Contents of this supplement

Tables

Table S1	Summary of data inputs and sources.	3
Table S2	Search strings used to identify studies of serotype distribution in pediatric acute respiratory infections.	5
Table S3	Studies included in meta-analysis of pneumococcal serotype distribution in nasopharyngeal samples from children with acute respiratory infections.	6
Table S4	Estimates of pneumococcal-attributable (any serotype) proportions of cases.	7
Table S5	Proportion of individuals with underlying health conditions qualifying for risk-based pneumococcal conjugate vaccine recommendations.	8
Table S6	Pneumococcal conjugate vaccine effectiveness (VE) inputs	9
Table S7	Estimated proportion of all pneumococcal isolates belonging to each serotype., by age group and clinical syndrome.	10
Table S8	Estimated proportion of pneumococcal infections and number of cases potentially preventable by 21-valent pneumococcal conjugate vaccine (PCV21) in children with risk factors for pneumococcal disease	15
Table S9	Proportion of pneumococcal infections due to serotypes targeted by pneumococcal conjugate vaccine products assuming complete serotype 15B and 15C cross-protection and comparison with primary analysis estimates among children aged 0–17 years.	16
Table S10	Proportion of pneumococcal infections due to serotypes targeted by pneumococcal conjugate vaccine products assuming complete serotype 15B and 15C cross-protection and comparison with primary analysis estimates among adults aged ≥18 years.	17
Table S11	Estimated national burdens of invasive pneumococcal disease in 2019.	19
Table S12	Estimated national burdens of all-cause outpatient AOM, pneumonia, and sinusitis visits among outpatients in 2019.	20
Table S13	Estimated national burdens of all-cause inpatient pneumonia in 2019.	21

Table S14	Preventable proportion of pneumococcal infections due to serotypes targeted by pneumococcal conjugate vaccines excluding PCV13 serotypes + 6C.	22
Table S15	Preventable proportion of pneumococcal infections due to serotypes targeted by pneumococcal conjugate vaccines excluding PCV13 serotypes + 6C assuming complete serotype 15B and 15C cross-protection.	24
Table S16	Estimated number of cases potentially preventable by pneumococcal conjugate vaccines in children 0–17 years.	26
Table S17	Estimated number of cases potentially preventable by pneumococcal conjugate vaccines assuming complete serotype 15B and 15C cross-protection in children aged 0–17 years.	29
Table S18	Estimated number of cases potentially preventable by pneumococcal conjugate vaccines in adults aged ≥18 years.	32
Table S19	Estimated number of cases potentially preventable by pneumococcal conjugate vaccines assuming complete serotype 15B and 15C cross-protection in adults aged ≥18 years.	35

Figures

Figure S1	Evidence from comparative studies for associations of vaccine immunogenicity with serotype-specific protection against colonization.	38

Text

Text S1	Supplemental references.	39

Table S1: Summary of data inputs and sources.

Measure	Condition	Age group	Source
Serotype distribution	AOM	Children 0–17 years	Meta-analysis of published studies (**Table S3**)
Serotype distribution	Non-bacteremic pneumonia	Children 0–17 years	Meta-analysis of published studies (**Table S3**)[a]
Serotype distribution	Sinusitis	Children and adults ≥5 years[b]	Meta-analysis of published studies (**Table S3**)[a]
Serotype distribution	Non-bacteremic pneumonia	Adults ≥18 years	PNEUMO study of hospitalized adults using 30-serotype SSUAD[1] + 2015–2019 ABCs[2] [c]
Serotype distribution	IPD	All ages	2015–2019 ABCs[2]
Proportion of cases that are pneumococcal-attributable	AOM, sinusitis, non-bacteremic pneumonia	All ages	Published studies (**Table S4**)
Annual number and incidence of all-cause outpatient-managed visits	AOM	Children 0–17 years	2016, 2019 NAMCS/NHAMCS + 2016–2019 MarketScan[d]
Annual number and incidence of all-cause outpatient-managed visits	Non-bacteremic pneumonia	Adults ≥18 years	2016, 2019 NAMCS/NHAMCS + 2016–2019 MarketScan[d]
Annual number and incidence of all-cause outpatient-managed visits	Sinusitis	Children and adults ≥5 years[b]	2016, 2019 NAMCS/NHAMCS + 2016–2019 MarketScan[d]
Annual number and incidence of all-cause hospitalizations	Non-bacteremic pneumonia	All ages	2019 NIS[e]
Annual number and incidence of cases	IPD	All ages	2019 ABCs
Proportion of cases occurring among individuals at increased risk of pneumococcal disease	Non-bacteremic pneumonia, IPD	Children and adults 5–64 years	Published studies (**Table S5**)

Measure	Condition	Age group	Source
PCV effectiveness against vaccine-serotype disease	All conditions	All ages	Published studies (**Table S6**)

Abbreviations: AOM – acute otitis media; PNEUMO -- Pneumococcal pNeumonia Epidemiology, Urine serotyping, and Mental Outcomes; SSUAD – serotype-specific urinary antigen detection; ABCs – Active Bacterial Core surveillance; IPD – invasive pneumococcal disease; NAMCS – National Ambulatory Medical Care Survey; NHAMCS – National Hospital Ambulatory Medical Care Survey; NIS – National Inpatient Sample.

[a] Based on meta-analysis of studies of serotype distribution in pediatric AOM and pneumonia.

[b] Sinusitis only considered in children ≥5 years due to incomplete sinusitis development and low counts of sinusitis diagnoses in NAMCS/NHAMCS precluding analysis in young ages.

[c] SSUAD data used for 30 SSUAD-type serotypes with additional serotype prevalence inferred from ABCs IPD data.

[d] NAMCS/NHAMCS and MarketScan methods.

[e] NIS minus bacteremic pneumonia from ABCs.

Table S2: Search strings used to identify studies of serotype distribution in pediatric acute respiratory infections.

String name	Search terms
**S1**	“((((*Streptococcus pneumoniae*) OR (pneumococcus)) OR (pneumococcal)) OR (*S. pneumoniae*)) AND (serotype) AND ((non-invasive) OR (outpatient))”
**S2**	“((((*Streptococcus pneumoniae*) OR (pneumococcus)) OR (pneumococcal)) OR (*S. pneumoniae*)) AND (serotype) AND ((otitis media) OR (otitis) OR (acute otitis media) OR (pneumonia) OR (sinusitis))”

Table S3: Studies included in meta-analysis of pneumococcal serotype distribution in nasopharyngeal samples from children with acute respiratory infections.

Reference	Study years	Study country	Participant ages	Condition	Serotyping method(s)	N isolates
Chen 2020[3]	2016–2018	Taiwan	2–60 months	AOM	Latex agglutination; Quellung reaction;[a] PCR	42
Kaur JID 2024[4]	2011–2019	United States	6–36 months	AOM	Quellung reaction[a]	400
Kaur PIDJ 2024[5]	2021–2023	United States	6–36 months	AOM	Quellung reaction[a]	79
Lewnard 2021[6,b]	2009–2018	Israel	0–59 months	Pneumonia	Quellung reaction[a]	117
Martin 2018[7]	2012–2014	United States	6–23 months	AOM	Quellung reaction[a]	113
Rybak 2023[8]	2010–2022	France	6–24 months	AOM	Latex agglutination	5759

Abbreviations: N – number; AOM – acute otitis media; PCR – polymerase chain reaction; JID – Journal of Infectious Diseases; PIDJ – Pediatric Infectious Diseases Journal.

[a] Serum Staten Institute, Copenhagen, Denmark.

[b] Only samples from Jewish children included (Bedouin children excluded). Although a small proportion of samples are from the pre-PCV13 period, we included this study as few others with serotype-specific estimates were available for pneumonia or other lower respiratory tract infections.

Table S4: Estimates of pneumococcal-attributable (any serotype) proportions of cases.

Condition, age group	Reported proportion of cases attributable to *S. pneumoniae*, % (95% CI)	Source
AOM, children 0–17 years	19.4 (16.8, 22.2)	King 2024[9]
Sinusitis, children 0–17 years	45.7 (28.4, 63.7)	King 2024[9]
Non-bacteremic pneumonia, children 0–17 years	27.1 (17.4, 38.5)	King 2024[9]
Sinusitis, adults ≥18 years[b]	45.7 (28.4, 63.7)	King 2024[9]
Non-bacteremic pneumonia,[c] adults 18–49 years	9.9 (8.1, 12.1)	Self 2024[1]
Non-bacteremic pneumonia,[c] adults 50–64 years	14.7 (12.5, 17.0)	Self 2024[1]
Non-bacteremic pneumonia,[c] adults ≥65 years	11.4 (9.6, 13.3)	Self 2024[1]

Abbreviations: CI – confidence interval; AOM – acute otitis media.

[a] Estimates presented are those reported. To propagate uncertainty when including multiple inputs in estimates of preventable burdens, we fit the reported pneumococcal-attributable percent values to gamma distributions and sampled 4,900,000 draws from these distributions.

[b] The percent of adult sinusitis due to pneumococcus assumed to be consistent with the pneumococcal-attributable percent in pediatric sinusitis.

[c] Original study included both bacteremic and non-bacteremic pneumonia cases in their case definition.

Table S5: Proportion of individuals with underlying health conditions qualifying for risk-based pneumococcal conjugate vaccine recommendations.

Condition	Setting	Age group	Estimated proportion, % (95% CI)[a,b]	Source
Pneumococcal pneumonia[c]				
	All	5–17 years	21.6 (20.3, 23.0)	Pelton 2014[10]
	Outpatient[d]	18–49 years	43.8 (43.0, 44.5)	Grant 2023[11]
	Inpatient[d]	18–49 years	58.5 (57.4, 59.5)	Grant 2023[11]
IPD				
	All	5–17 years	31.0 (25.9, 36.3)	Pelton 2014[10]
	All	18–49 years	68.3 (63.8, 72.5)	Grant 2023[11]

Abbreviations: PCV – pneumococcal conjugate vaccine; CI – confidence interval; IPD – invasive pneumococcal disease.
[a] PCV15, PCV20, or PCV21 in adults and PCV15 or PCV20 in children.[12–15]
[b] Obtained by fitting the no. cases among individuals with underlying health conditions / total no. cases and corresponding standard error to beta distributions and sampling 4,900,000 draws.
[c] Pneumococcal pneumonia estimates applied to all respiratory infections (acute otitis media, sinusitis) in corresponding age groups.
[d] Estimated using the proportion of cases occurring in each setting for all adults 18–64 years (Grant 2023[11] Supplemental Table 6) and number of cases in each age group (Grant 2023[11] Supplemental Table 5).

Table S6: Pneumococcal conjugate vaccine effectiveness (VE) inputs.

Condition	PCV	Reported PCV effectiveness, % (95% CI)[a,b]	Source
Pediatric AOM	PCV7	57.0 (44.0, 67.0)	Eskola 2001[16]
Pediatric sinusitis[c]	PCV7	57.0 (44.0, 67.0)	Eskola 2001[16]
Pediatric non-bacteremic pneumonia	PCV13	53.2 (45.4, 57.2)	Estimated[d]
Pediatric IPD	PCV13	90.2 (75.4, 96.1)	Andrejko 2024[17]
Adult sinusitis[c]	PCV7	57.0 (44.0, 67.0)	Eskola 2001[16]
Adult non-bacteremic pneumonia	PCV13	45.0 (14.2, 65.3)	Bonten 2015[18]
Adult IPD	PCV13	75.0 (41.4, 90.8)	Bonten 2015[18]

Abbreviations: CI – confidence interval; PCV – pneumococcal conjugate vaccine; AOM – acute otitis media; IPD – invasive pneumococcal disease.

[a] Estimates presented are those reported in previous studies. To propagate uncertainty when including multiple inputs in estimates of preventable burdens, we fit the reported VE values to beta distributions and sampled 4,900,000 draws from these distributions.

[b] VE against vaccine-type disease.

[c] Sinusitis VE considered to be consistent with that for AOM; no studies of PCV VE against sinusitis are available.

[d] Obtained by applying the ratio of PCV13 VE in adult CAP to adult IPD (45:75) to PCV13 VE against pediatric IPD estimates consistent with prior studies.[19]

Table S7: Estimated proportion of all pneumococcal isolates belonging to each serotype, by age group and clinical syndrome.

	Estimated proportion of all pneumococcal isolates, % (95% CI)[a]								
**Sero-type**	*AOM 0–17 years*	*Pneumonia,[b] sinusitis 0–17 years*	*Pneumonia[b] 18–49 years*	*Pneumonia[b] 50–64 years*	*Pneumonia[b] ≥65 years*	*IPD 0–17 years*	*IPD 18–49 years*	*IPD 50–64 years*	*IPD ≥65 years*
**23B**	9.4 (8.7, 10.2)	9.3 (8.6, 10.0)	6.0 (2.4, 12.4)	2.2 (0.6, 5.3)	1.7 (0.3, 4.8)	7.7 (6.2, 9.4)	3.4 (2.7, 4.1)	3.3 (2.8, 3.9)	2.9 (2.5, 3.4)
**11A**	9.0 (8.3, 9.7)	8.8 (8.2, 9.6)	2.0 (0.4, 6.6)	4.3 (2.0, 8.4)	4.0 (1.8, 8.3)	2.1 (1.4, 3.2)	3.1 (2.5, 3.8)	3.7 (3.2, 4.3)	4.2 (3.7, 4.7)
**15A**	7.8 (7.2, 8.5)	7.8 (7.1, 8.4)	0 (0, 0)	2.7 (0.8, 6.1)	0 (0, 0)	4.2 (3.1, 5.5)	2.8 (2.2, 3.5)	3.9 (3.4, 4.5)	5.7 (5.1, 6.3)
**35B**	7.7 (7.0, 8.3)	7.6 (6.9, 8.2)	7.4 (3.0, 14.3)	3.6 (1.3, 7.4)	3.9 (1.4, 8.1)	5.3 (4.0, 6.7)	3.0 (2.4, 3.7)	3.5 (3, 4)	6.1 (5.5, 6.8)
**23A**	5.9 (5.4, 6.5)	5.9 (5.3, 6.5)	4.2 (1.3, 9.4)	2.4 (0.7, 5.5)	5.5 (2.6, 9.9)	2.7 (1.9, 3.8)	3.9 (3.2, 4.7)	4.5 (3.9, 5.1)	6.0 (5.4, 6.6)
**21**	5 (4.5, 5.5)	4.9 (4.4, 5.5)	0 (0, 2.0)	0.4 (0.1, 2.6)	0 (0, 1.3)	2.6 (1.8, 3.7)	0.2 (0.1, 0.5)	0.2 (0.1, 0.4)	0.2 (0.1, 0.3)
**19A**	4.3 (3.8, 4.8)	4.3 (3.8, 4.8)	8.7 (3.8, 15.7)	5 (2.2, 9.2)	4.1 (1.5, 8.4)	6.4 (5, 8)	3.7 (3, 4.4)	3.7 (3.2, 4.2)	2.9 (2.5, 3.3)
**35F**	4.3 (3.8, 4.8)	4.2 (3.8, 4.8)	0.9 (0, 4.2)	2.4 (0.6, 6.0)	2 (0.3, 5.4)	1.7 (1, 2.6)	0.9 (0.6, 1.3)	1.5 (1.1, 1.8)	1.5 (1.2, 1.9)
**10A**	4.0 (3.5, 4.5)	4.0 (3.5, 4.5)	0 (0, 0.1)	0 (0, 0.1)	0.5 (0, 2.2)	3.4 (2.4, 4.5)	1.8 (1.4, 2.3)	2.0 (1.6, 2.4)	1.4 (1.1, 1.7)
**24F**	3.0 (2.6, 3.4)	2.9 (2.5, 3.4)	0.4 (0, 2.7)	0.9 (0.1, 3.0)	0 (0, 0)	0 (0, 0)	0 (0, 0)	0 (0, 0)	0 (0, 0)
**19F**	2.9 (2.5, 3.3)	2.9 (2.5, 3.4)	0.4 (0, 3.5)	5.4 (2.5, 9.5)	4.6 (1.8, 8.7)	7.7 (6.2, 9.4)	3.4 (2.7, 4.1)	3.0 (2.6, 3.5)	2.4 (2.0, 2.8)
**NT**	2.3 (2.0, 2.7)	2.4 (2.1, 2.8)	0.1 (0, 1.6)	0 (0, 1.0)	0 (0, 1.2)	0.3 (0.1, 0.7)	0.1 (0, 0.2)	0.1 (0.1, 0.2)	0.1 (0.0, 0.2)
**22F**	2.3 (2.0, 2.7)	2.3 (1.9, 2.7)	7.2 (2.7, 13.7)	9.9 (5.8, 15.3)	5.3 (2.1, 9.9)	8.9 (7.3, 10.7)	9.4 (8.3, 10.5)	10.3 (9.4, 11.2)	10.9 (10.1, 11.7)
**6C[c]**	2.3 (1.9, 2.7)	2.3 (1.9, 2.6)	-	-	-	1.0 (0.5, 1.7)	2.6 (2.1, 3.3)	2.4 (2.0, 2.9)	3.7 (3.3, 4.3)
**15C**	11.4 (10.6, 12.3)	11.4 (10.5, 12.2)	1.6 (0.3, 5.5)	0.3 (0, 2.1)	1.7 (0.5, 4.6)	5.9 (4.6, 7.4)	0.9 (0.6, 1.3)	0.8 (0.6, 1.1)	1.2 (1.0, 1.5)
**33F**	1.9 (1.6, 2.2)	1.9 (1.5, 2.2)	0.6 (0, 4.1)	1 (0.1, 3.5)	0.4 (0, 2.7)	6.7 (5.3, 8.3)	3.8 (3.1, 4.5)	3.3 (2.8, 3.8)	4.4 (3.9, 5.0)
**31**	1.7 (1.4, 2.0)	1.7 (1.4, 2.0)	0 (0, 0)	4.0 (1.6, 8.2)	2.3 (0.6, 6.0)	0.6 (0.2, 1.2)	1.6 (1.2, 2.1)	1.9 (1.6, 2.4)	2.8 (2.4, 3.3)
**17F**	1.7 (1.4, 2.0)	1.6 (1.3, 2.0)	3.0 (0.5, 7.9)	1.7 (0.3, 4.6)	3.3 (1.0, 7.2)	0.6 (0.3, 1.3)	1.7 (1.2, 2.2)	1.3 (1.0, 1.6)	1.3 (1.0, 1.6)
**16F**	1.6 (1.3, 1.9)	1.6 (1.3, 2.0)	1.3 (0.2, 5.8)	0.2 (0, 1.9)	3.7 (1.4, 7.9)	3.1 (2.1, 4.2)	3.0 (2.4, 3.7)	3.8 (3.2, 4.3)	4.4 (3.9, 5.0)
**15B**	1.6 (1.3, 1.9)	1.5 (1.2, 1.9)	1.7 (0.3, 5.8)	1.7 (0.4, 4.4)	3.2 (1.1, 6.9)	3.9 (2.9, 5.2)	1.3 (1.0, 1.8)	1.1 (0.8, 1.4)	2.0 (1.7, 2.4)
**3**	1.4 (1.1, 1.7)	1.4 (1.1, 1.7)	9.3 (4.7, 16.5)	13.0 (8.6, 18.8)	10.8 (6.6, 16.6)	9.0 (7.4, 10.9)	11.2 (10.1, 12.4)	14.3 (13.3, 15.3)	15.8 (14.9, 16.8)
**9N**	1.2 (0.9, 1.5)	1.2 (1.0, 1.5)	6.5 (2.5, 12.9)	5.6 (2.7, 10.0)	1.9 (0.4, 5.4)	1.9 (1.2, 2.8)	7.4 (6.5, 8.4)	6.9 (6.2, 7.6)	4.0 (3.5, 4.6)

	Estimated proportion of all pneumococcal isolates, % (95% CI)[a]								
**Sero-type**	*AOM 0–17 years*	*Pneumonia,[b] sinusitis 0–17 years*	*Pneumonia[b] 18–49 years*	*Pneumonia[b] 50–64 years*	*Pneumonia[b] ≥65 years*	*IPD 0–17 years*	*IPD 18–49 years*	*IPD 50–64 years*	*IPD ≥65 years*
**29**	0.9 (0.7, 1.1)	0.9 (0.7, 1.1)	0 (0, 0.9)	0 (0, 0.7)	0 (0, 0.7)	0 (0, 0.1)	0 (0, 0)	0 (0, 0)	0 (0, 0)
**11C**	0.9 (0.5, 1.5)	0.9 (0.5, 1.4)	0 (0, 1.0)	0 (0, 0.8)	0 (0, 0.7)	0 (0, 0.1)	0 (0, 0)	0 (0, 0)	0 (0, 0)
**35D**	0.7 (0.4, 1.3)	0.7 (0.4, 1.3)	0 (0, 0.1)	0 (0, 0.1)	0 (0, 0.1)	0.1 (0, 0.4)	0 (0, 0.1)	0.1 (0, 0.2)	0.1 (0, 0.2)
**6A[c]**	0.6 (0.4, 0.8)	0.4 (0.2, 0.6)	2.0 (0.3, 5.9)	0.6 (0.1, 2.5)	4.8 (1.9, 8.9)	0 (0, 0.1)	0.1 (0, 0.3)	0.1 (0.1, 0.3)	0.1 (0.1, 0.2)
**40**	0.4 (0.2, 1)	0.4 (0.2, 0.9)	0 (0, 0.6)	0 (0, 0.5)	0 (0, 0.5)	0 (0, 0.1)	0 (0, 0)	0 (0, 0)	0 (0, 0)
**11D**	0.3 (0.1, 0.7)	0.3 (0.1, 0.7)	0 (0, 0.8)	0 (0, 0.5)	0 (0, 0.6)	0 (0, 0.1)	0 (0, 0)	0 (0, 0)	0 (0, 0)
**34**	0.3 (0.1, 0.7)	0.3 (0.1, 0.6)	1.5 (0.3, 6.1)	1.5 (0.4, 4.6)	2.4 (0.7, 6.2)	0.9 (0.4, 1.6)	1.1 (0.8, 1.6)	1.3 (1.0, 1.6)	1.6 (1.3, 2.0)
**38**	0.3 (0, 0.7)	0.3 (0, 0.7)	0.3 (0, 4.1)	1.4 (0.2, 4.5)	1.5 (0.3, 4.9)	2.4 (1.6, 3.4)	0.7 (0.4, 1.0)	1.0 (0.8, 1.4)	1.4 (1.1, 1.8)
**23F**	0.2 (0.1, 0.4)	0.1 (0, 0.2)	0 (0, 0.1)	0 (0, 0.1)	0 (0, 0.1)	0 (0, 0.3)	0.1 (0, 0.2)	0.1 (0, 0.1)	0 (0, 0.1)
**7C**	0.2 (0, 0.6)	0.2 (0, 0.6)	1.8 (0.5, 5.3)	2.3 (0.9, 5.3)	3.2 (1.4, 6.7)	2.0 (1.2, 2.9)	1.6 (1.2, 2.1)	1.5 (1.2, 1.9)	2.4 (2.0, 2.8)
**13**	0.2 (0, 0.6)	0.2 (0, 0.5)	0.8 (0.1, 3.9)	0.5 (0, 2.2)	0 (0, 0.8)	0.1 (0, 0.3)	0.4 (0.2, 0.7)	0.2 (0.1, 0.3)	0.1 (0.1, 0.2)
**25A**	0.1 (0, 0.5)	0.1 (0, 0.5)	0 (0, 0)	0 (0, 0)	0 (0, 0)	0 (0, 0)	0 (0, 0)	0 (0, 0)	0 (0, 0)
**28F**	0.1 (0, 0.5)	0.1 (0, 0.5)	0 (0, 0)	0 (0, 0)	0 (0, 0)	0 (0, 0)	0 (0, 0)	0 (0, 0)	0 (0, 0)
**37**	0.1 (0, 0.5)	0.1 (0, 0.4)	0 (0, 0.1)	0 (0, 0.1)	0 (0, 0.1)	0 (0, 0)	0.1 (0, 0.3)	0.1 (0, 0.2)	0.1 (0, 0.2)
**7A**	0.1 (0, 0.5)	0.1 (0, 0.4)	0 (0, 1.0)	0 (0, 0.5)	0 (0, 0.7)	0 (0, 0.1)	0 (0, 0)	0 (0, 0)	0 (0, 0)
**15F**	0.1 (0, 0.4)	0.1 (0, 0.4)	0 (0, 0.9)	0 (0, 0.5)	0 (0, 0.5)	0 (0, 0.4)	0 (0, 0.1)	0 (0, 0)	0 (0, 0)
**19B**	0.1 (0, 0.4)	0.1 (0, 0.4)	0 (0, 0.3)	0 (0, 0.2)	0 (0, 0.2)	0 (0, 0.1)	0 (0, 0)	0 (0, 0)	0 (0, 0)
**33D**	0.1 (0, 0.4)	0.1 (0, 0.3)	0 (0, 0.9)	0 (0, 0.7)	0 (0, 0.8)	0 (0, 0.1)	0 (0, 0.1)	0 (0, 0)	0 (0, 0)
**39**	0.1 (0, 0.3)	0.1 (0, 0.3)	0 (0, 0.1)	0 (0, 0.4)	0 (0, 0.1)	0 (0, 0)	0 (0, 0)	0 (0, 0)	0 (0, 0)
**4**	0.1 (0, 0.3)	0 (0, 0.1)	0 (0, 0.4)	1.1 (0.1, 3.1)	0.4 (0, 2.1)	0.3 (0.1, 0.8)	5.6 (4.7, 6.4)	2.7 (2.3, 3.2)	0.6 (0.4, 0.9)
**14**	0 (0, 0)	0.4 (0.3, 0.6)	0 (0, 1.8)	0.3 (0, 2.7)	0 (0, 1.2)	0 (0, 0.2)	0.1 (0, 0.2)	0.1 (0, 0.2)	0.2 (0.1, 0.3)
**12F**	0 (0, 0)	0.1 (0, 0.3)	0.6 (0.1, 3.4)	0 (0, 0.7)	0 (0, 0.8)	2.6 (1.8, 3.7)	6.0 (5.2, 6.9)	5.0 (4.4, 5.7)	2.3 (1.9, 2.7)
**7F**	0 (0, 0)	0.1 (0, 0.3)	0.2 (0, 4.3)	2.0 (0.6, 5.4)	3.7 (1.4, 7.9)	0.7 (0.3, 1.3)	2.5 (1.9, 3.1)	1.6 (1.2, 2.0)	0.6 (0.5, 0.9)
**10C**	0 (0, 0)	0 (0, 0)	0 (0, 0.4)	0 (0, 0.2)	0 (0, 0.3)	0 (0, 0)	0 (0, 0)	0 (0, 0)	0 (0, 0)
**10F**	0 (0, 0)	0 (0, 0)	0 (0, 1.0)	0 (0, 0.6)	0 (0, 0.6)	0.2 (0, 0.5)	0 (0, 0.1)	0.1 (0, 0.2)	0.1 (0, 0.2)
**11B**	0 (0, 0)	0 (0, 0)	0 (0, 1.2)	0 (0, 0.9)	0 (0, 1.0)	0 (0, 0.2)	0 (0, 0.1)	0 (0, 0)	0 (0, 0)

Sero-type	Estimated proportion of all pneumococcal isolates, % (95% CI)[a]								
	AOM 0–17 years	*Pneumonia,[b] sinusitis 0–17 years*	*Pneumonia[b] 18–49 years*	*Pneumonia [b] 50–64 years*	*Pneumonia [b] ≥65 years*	*IPD 0–17 years*	*IPD 18–49 years*	*IPD 50–64 years*	*IPD ≥65 years*
**11E**	0 (0, 0)	0 (0, 0)	0 (0, 0.5)	0 (0, 0.4)	0 (0, 0.3)	0 (0, 0.1)	0 (0, 0)	0 (0, 0)	0 (0, 0)
**12A**	0 (0, 0)	0 (0, 0)	0 (0, 0)	0 (0, 0)	0 (0, 0)	0 (0, 0)	0 (0, 0)	0 (0, 0)	0 (0, 0)
**12B**	0 (0, 0)	0 (0, 0)	0 (0, 0.3)	0 (0, 0.3)	0 (0, 0.2)	0 (0, 0)	0 (0, 0)	0 (0, 0)	0 (0, 0)
**16A**	0 (0, 0)	0 (0, 0)	0 (0, 0)	0 (0, 0)	0 (0, 0)	0 (0, 0)	0 (0, 0)	0 (0, 0)	0 (0, 0)
**17A**	0 (0, 0)	0 (0, 0)	0 (0, 1.8)	0 (0, 1.2)	0 (0, 1.1)	0 (0, 0.2)	0 (0, 0.1)	0 (0, 0)	0 (0, 0)
**18A**	0 (0, 0)	0 (0, 0)	0 (0, 0)	0 (0, 0)	0 (0, 0)	0 (0, 0)	0 (0, 0.2)	0 (0, 0.1)	0 (0, 0)
**18F**	0 (0, 0)	0 (0, 0)	0 (0, 1.5)	0 (0, 1.0)	0 (0, 1.1)	0 (0, 0.1)	0 (0, 0.1)	0 (0, 0)	0 (0, 0)
**19C**	0 (0, 0)	0 (0, 0)	0 (0, 0)	0 (0, 0)	0 (0, 0)	0 (0, 0)	0 (0, 0)	0 (0, 0)	0 (0, 0)
**27**	0 (0, 0)	0 (0, 0)	0 (0, 0)	0 (0, 0)	0 (0, 0)	0 (0, 0)	0 (0, 0)	0 (0, 0)	0 (0, 0)
**28A**	0 (0, 0)	0 (0, 0)	0 (0, 0.1)	0.2 (0, 1.8)	0.2 (0, 1.9)	0 (0, 0.3)	0.2 (0.1, 0.3)	0.2 (0.1, 0.4)	0.3 (0.2, 0.4)
**32A**	0 (0, 0)	0 (0, 0)	0 (0, 0.2)	0 (0, 0.3)	0 (0, 0.4)	0 (0, 0)	0 (0, 0)	0 (0, 0)	0 (0, 0)
**33A**	0 (0, 0)	0 (0, 0)	0 (0, 0.1)	0 (0, 0.1)	0 (0, 0.1)	0.1 (0, 0.3)	0 (0, 0.1)	0 (0, 0)	0 (0, 0)
**35A**	0 (0, 0)	0 (0, 0)	0 (0, 0.2)	0 (0, 0.2)	0 (0, 0.1)	0 (0, 0)	0 (0, 0)	0 (0, 0)	0 (0, 0)
**36**	0 (0, 0)	0 (0, 0)	0 (0, 0)	0 (0, 0)	0 (0, 0)	0 (0, 0)	0 (0, 0)	0 (0, 0)	0 (0, 0)
**47F**	0 (0, 0)	0 (0, 0)	0 (0, 0.7)	0 (0, 0.3)	0 (0, 0.4)	0 (0, 0)	0 (0, 0)	0 (0, 0)	0 (0, 0)
**6D**	0 (0, 0)	0 (0, 0)	0 (0, 0.6)	0 (0, 0.4)	0 (0, 0.3)	0 (0, 0.1)	0 (0, 0)	0 (0, 0)	0 (0, 0.1)
**6H**	0 (0, 0)	0 (0, 0)	0.1 (0, 2.1)	0.1 (0, 1.1)	0.1 (0, 1.2)	0 (0, 0.2)	0 (0, 0.1)	0 (0, 0)	0 (0, 0)
**8**	0 (0, 0)	0 (0, 0)	2.7 (0.6, 7.4)	1.6 (0.4, 4.3)	3.1 (1.1, 6.9)	1.9 (1.2, 2.9)	7 (6.1, 8.0)	5.2 (4.6, 5.9)	2.9 (2.5, 3.4)
**24A**	0 (0, 0)	0 (0, 0.1)	0 (0, 0.1)	0 (0, 0.1)	0 (0, 0.1)	0 (0, 0)	0 (0, 0)	0 (0, 0)	0 (0, 0)
**20A**	0 (0, 0.3)	0 (0, 0.3)	2.5 (0.6, 7.2)	2.1 (0.7, 5.2)	0.9 (0.1, 3.4)	0 (0, 0)	0 (0, 0)	0 (0, 0)	0 (0, 0)
**44**	0 (0, 0.3)	0 (0, 0.3)	0 (0, 0)	0 (0, 0)	0 (0, 0)	0 (0, 0)	0 (0, 0)	0 (0, 0)	0 (0, 0)
**48**	0 (0, 0.3)	0 (0, 0.3)	0 (0, 1.1)	0 (0, 0.7)	0 (0, 0.6)	0 (0, 0.1)	0 (0, 0)	0 (0, 0)	0 (0, 0)
**9L**	0 (0, 0.3)	0 (0, 0.3)	0 (0, 0.9)	0 (0, 0.7)	0 (0, 0.6)	0 (0, 0.1)	0 (0, 0)	0 (0, 0.1)	0 (0, 0.1)
**33C**	0 (0, 0.3)	0 (0, 0.2)	0 (0, 0.3)	0 (0, 0.2)	0 (0, 0.2)	0 (0, 0.1)	0 (0, 0)	0 (0, 0)	0 (0, 0)
**6B**	0 (0, 0.2)	0.1 (0, 0.2)	0 (0, 0.8)	1.1 (0.1, 3.5)	0.5 (0, 2.6)	0.2 (0, 0.5)	0.1 (0, 0.2)	0.2 (0.1, 0.3)	0.2 (0.1, 0.4)
**9V**	0 (0, 0.2)	0.1 (0, 0.2)	2.9 (0.7, 7.1)	0.4 (0, 2)	1.1 (0.2, 3.4)	0.1 (0, 0.3)	0.1 (0, 0.3)	0.3 (0.1, 0.4)	0.1 (0, 0.2)

	Estimated proportion of all pneumococcal isolates, % (95% CI)[a]								
**Sero-type**	*AOM 0–17 years*	*Pneumonia,[b] sinusitis 0–17 years*	*Pneumonia[b] 18–49 years*	*Pneumonia [b] 50–64 years*	*Pneumonia [b] ≥65 years*	*IPD 0–17 years*	*IPD 18–49 years*	*IPD 50–64 years*	*IPD ≥65 years*
**10D**	0 (0, 0.2)	0 (0, 0.2)	0 (0, 2.5)	0 (0, 1.5)	0 (0, 1.5)	0 (0, 0.2)	0 (0, 0.1)	0 (0, 0.1)	0 (0, 0)
**20B**	0 (0, 0.2)	0 (0, 0.2)	7.2 (3, 13.9)	6.7 (3.4, 11.3)	3.1 (1, 7)	0.8 (0.4, 1.5)	4.3 (3.6, 5.1)	4.4 (3.8, 5)	2.4 (2, 2.8)
**25F**	0 (0, 0.2)	0 (0, 0.2)	0 (0, 0.4)	0 (0, 0.2)	0 (0, 0.3)	0 (0, 0)	0 (0, 0)	0 (0, 0)	0 (0, 0)
**32F**	0 (0, 0.2)	0 (0, 0.2)	0 (0, 1.2)	0 (0, 0.9)	0 (0, 0.9)	0 (0, 0.1)	0 (0, 0)	0 (0, 0)	0 (0, 0)
**33B**	0 (0, 0.2)	0 (0, 0.2)	0 (0, 1)	0 (0, 0.5)	0 (0, 0.5)	0 (0, 0.1)	0 (0, 0)	0 (0, 0)	0 (0, 0)
**35C**	0 (0, 0.2)	0 (0, 0.2)	0 (0, 0.6)	0 (0, 0.5)	0 (0, 0.4)	0 (0, 0.1)	0 (0, 0)	0 (0, 0)	0 (0, 0)
**42**	0 (0, 0.2)	0 (0, 0.2)	0 (0, 0.2)	0 (0, 0.1)	0 (0, 0.1)	0 (0, 0)	0 (0, 0)	0 (0, 0)	0 (0, 0)
**7D**	0 (0, 0.2)	0 (0, 0.2)	0 (0, 0)	0 (0, 0)	0 (0, 0)	0 (0, 0)	0 (0, 0)	0 (0, 0)	0 (0, 0)
**9A**	0 (0, 0.2)	0 (0, 0.2)	0 (0, 0.4)	0 (0, 0.2)	0 (0, 0.2)	0 (0, 0)	0 (0, 0)	0 (0, 0)	0 (0, 0)
**24C**	0 (0, 0.2)	0 (0, 0.1)	0 (0, 0.3)	0 (0, 0.2)	0 (0, 0.2)	0 (0, 0)	0 (0, 0)	0 (0, 0)	0 (0, 0)
**43**	0 (0, 0.2)	0 (0, 0.1)	0 (0, 1.2)	0 (0, 0.8)	0 (0, 1)	0 (0, 0.1)	0 (0, 0.1)	0 (0, 0)	0 (0, 0)
**1**	0 (0, 0.1)	0.3 (0.1, 0.5)	0 (0, 0.7)	0.5 (0, 2.3)	4 (1.4, 8.3)	0 (0, 0.1)	0.1 (0, 0.3)	0 (0, 0)	0 (0, 0)
**5**	0 (0, 0.1)	0.1 (0, 0.2)	1.7 (0.5, 5.2)	1 (0.3, 3)	1.8 (0.7, 4.4)	0 (0, 0)	0 (0, 0)	0 (0, 0.1)	0 (0, 0)
**18C**	0 (0, 0.1)	0 (0, 0)	0 (0, 1.3)	0 (0, 0.9)	0 (0, 0.9)	0.3 (0.1, 0.9)	0.1 (0, 0.2)	0.1 (0, 0.2)	0 (0, 0)
**10B**	0 (0, 0.1)	0 (0, 0.4)	0 (0, 0)	0 (0, 0)	0 (0, 0)	0 (0, 0)	0 (0, 0)	0 (0, 0)	0 (0, 0)
**11F**	0 (0, 0.1)	0 (0, 0.1)	0 (0, 0.9)	0 (0, 0.7)	0 (0, 0.6)	0 (0, 0.1)	0 (0, 0)	0 (0, 0)	0 (0, 0)
**18B**	0 (0, 0.1)	0 (0, 0.1)	0 (0, 0.1)	0 (0, 0.1)	0 (0, 0.1)	0 (0, 0)	0 (0, 0)	0 (0, 0)	0 (0, 0.1)
**2**	0.0 (0, 0.1)	0 (0, 0.1)	0 (0, 0.2)	0 (0, 0.2)	0 (0, 0.3)	0 (0, 0)	0 (0, 0)	0 (0, 0)	0 (0, 0)
**22A**	0 (0, 0.1)	0 (0, 0.1)	0 (0, 0.1)	0 (0, 0.1)	0 (0, 0.1)	0 (0, 0)	0 (0, 0)	0 (0, 0)	0 (0, 0)
**24B**	0 (0, 0.1)	0 (0, 0.1)	0.1 (0, 1.2)	0.1 (0, 0.8)	0.1 (0, 0.8)	0 (0, 0.1)	0 (0, 0)	0 (0, 0)	0 (0, 0)
**41A**	0 (0, 0.1)	0 (0, 0.1)	0 (0, 1.8)	0 (0, 1.2)	0 (0, 1.3)	0 (0, 0.2)	0 (0, 0.1)	0 (0, 0)	0 (0, 0)
**41F**	0 (0, 0.1)	0 (0, 0.1)	0 (0, 0.6)	0 (0, 0.3)	0 (0, 0.4)	0 (0, 0.1)	0 (0, 0)	0 (0, 0)	0 (0, 0)
**45**	0 (0, 0.1)	0 (0, 0.1)	0 (0, 0.2)	0 (0, 0.2)	0 (0, 0.2)	0 (0, 0)	0 (0, 0)	0 (0, 0)	0 (0, 0.1)
**46**	0 (0, 0.1)	0 (0, 0.1)	0 (0, 0)	0 (0, 0)	0 (0, 0)	0 (0, 0)	0 (0, 0)	0 (0, 0)	0 (0, 0)
**47A**	0 (0, 0.1)	0 (0, 0.1)	0 (0, 0.5)	0 (0, 0.3)	0 (0, 0.3)	0 (0, 0)	0 (0, 0)	0 (0, 0)	0 (0, 0)

Sero-type	Estimated proportion of all pneumococcal isolates, % (95% CI)[a]								
	AOM 0–17 years	*Pneumonia,[b] sinusitis 0–17 years*	*Pneumonia[b] 18–49 years*	*Pneumonia [b] 50–64 years*	*Pneumonia [b] ≥65 years*	*IPD 0–17 years*	*IPD 18–49 years*	*IPD 50–64 years*	*IPD ≥65 years*
**7B**	0 (0, 0.1)	0 (0, 0.1)	0 (0, 1.2)	0 (0, 0.9)	0 (0, 0.9)	0 (0, 0.1)	0 (0, 0)	0 (0, 0.1)	0 (0, 0)
**15D[d]**	-	-	0 (0, 0.1)	0 (0, 0.1)	0 (0, 0)	0 (0, 0)	0 (0, 0)	0 (0, 0)	0 (0, 0.1)

Abbreviations: CI – confidence interval; AOM – acute otitis media; IPD – invasive pneumococcal disease.

[a] Percent point estimates do not sum to 100% as reported due to rounding.

[b] Non-bacteremic pneumonia. Bacteremic pneumonia included in IPD.

[c] For adult pneumonia, estimates for 6A are for 6A and 6C combined due to serotype-specific urinary antigen detection assay cross-reactivity for these two serotypes.

[d] 15D has only been identified in invasive pneumococcal disease[20] and so was not considered for pediatric, non-invasive samples.

Table S8: Estimated proportion of pneumococcal infections and number of cases potentially preventable by 21-valent pneumococcal conjugate vaccine (PCV21) in children with risk factors for pneumococcal disease.

	Assuming no 15B and 15C cross-protection[a]		Assuming complete 15B and 15C cross-protection[a]	
**Condition**	*% pneumococcal isolates (95% CI)*[b]	*No. cases in thousands (95% CI)*[c]	*% pneumococcal isolates (95% CI)*[b]	*No. cases in thousands (95% CI)*[c]
AOM[d]	77.3 (76.1, 78.4)	64.7 (41.9, 97.4)	78.9 (77.8, 79.9)	66.2 (42.9, 99.6)
Sinusitis[d]	76.7 (75.5, 77.9)	116.6 (63.7, 195.3)	78.2 (77.2, 79.3)	119.2 (65.1, 199.6)
Pneumonia (outpatients)[e]	76.7 (75.5, 77.9)	19.8 (10.8, 33.6)	78.2 (77.2, 79.3)	20.3 (11.1, 34.3)
Pneumonia (inpatients)[e]	76.7 (75.5, 77.9)	0.5 (0.3, 0.8)	78.2 (77.2, 79.3)	0.5 (0.3, 0.8)
IPD	75.3 (72.6, 77.8)	0.1 (0.1, 0.1)	79.3 (76.7, 81.6)	0.1 (0.1, 0.2)

Abbreviations: CI – confidence interval; AOM – acute otitis media; IPD – invasive pneumococcal disease.

[a] Children aged 5–17 years for all conditions except non-bacteremic pneumonia managed in outpatient settings which includes children 2–17 years to ensure adequate sample size for National Ambulatory Medicare Care Survey projection validity.[21] PCV15 or PCV20 currently recommended for children 5–17 years with health conditions placing them at increased risk of pneumococcal disease.[12,13]

[b] Total percent of pneumococcal isolates not excluding PCV13 serotypes.

[c] Considering US population by age group[22] and assuming disease incidence rates as detailed in **Tables S11–S13**, pneumococcal-attributable disease proportions (**Table S4)**, preventable proportions of disease (excluding PCV13 serotypes), percents of pediatric pneumococcal cases occurring among children with risk-based PCV recommendations (**Table S5**), and PCV effectiveness (**Table S6).**

[d] Percent of cases occurring among children with risk-based indications for PCVs for pneumococcal AOM and sinusitis assumed to be consistent with pneumonia (**Table S5**).

[e] Non-bacteremic pneumonia. Bacteremic pneumonia classified as IPD.

Table S9: Proportion of pneumococcal infections due to serotypes targeted by pneumococcal conjugate vaccine products assuming complete serotype 15B and 15C cross-protection and comparison with primary analysis estimates among children aged 0–17 years.

		**PCV20[a,b]**		**PCV21[a,b]**		**PCV24[a,b,d]**		**PCV25[a,b]**		**PCV31[a,b]**	
**Condition**	**Age group**	*% of isolates (95% CI)*	*Percent change from primary (95% CI)* [c]	*% of isolates (95% CI)*	*Percent change from primary (95% CI)* [c]	*% of isolates (95% CI)*	*Percent change from primary (95% CI)* [c]	*% of isolates (95% CI)*	*Percent change from primary (95% CI)* [c]	*% of isolates (95% CI)*	*Percent change from primary (95% CI)* [c]
AOM	0–17 years	42.1 (40.9, 43.4)	37.3 (33.8, 41.0)	78.9 (77.8, 79.9)	2.0 (1.6, 2.5)	45.0 (43.8, 46.3)	34.1 (30.9, 37.4)	54.4 (53.2, 55.7)	26.6 (24.2, 29.2)	79.5 (78.4, 80.5)	16.8 (15.4, 18.3)
Sinusitis, pneumonia[e]	0–17 years	42.6 (41.4, 43.8)	36.4 (33.0, 39.9)	78.2 (77.2, 79.3)	2.0 (1.6, 2.4)	45.5 (44.3, 46.8)	33.3 (30.2, 36.5)	54.9 (53.7, 56.1)	26.1 (23.7, 28.5)	79.6 (78.6, 80.6)	16.6 (15.2, 18.1)
IPD	0–17 years	61.9 (58.9, 64.7)	10.6 (8.1, 13.6)	79.3 (76.7, 81.6)	5.2 (3.8, 7.0)	65.2 (62.4, 68.1)	10.0 (7.7, 12.8)	74.2 (71.4, 76.8)	8.7 (6.7, 11.1)	90.9 (89.0, 92.6)	7.0 (5.4, 8.9)

Abbreviations: PCV – pneumococcal conjugate vaccine; CI – confidence interval; AOM – acute otitis media; IPD – invasive pneumococcal disease.

[a] PCV20 includes serotypes 1, 3, 4, 5, 6A/C, 6B, 7F, 8, 9V, 10A, 11A, 12F, 14, 15B, 18C, 19F, 19A, 22F, 23F, 33F. PCV24 includes serotypes 1, 2, 3, 4, 5, 6A/C, 6B, 7F, 8, 9N, 9V, 10A, 11A, 12F, 14, 15B, 17F, 18C, 19F, 19A, 20B, 22F, 23F, 33F. PCV25 includes serotypes 1, 2, 3, 4, 5, 6B, 6A/C, 7F, 8, 9N, 9V, 10A, 12F, 14, 15A, 15B, 16F, 18C, 19F, 19A, 22F, 23F, 24F, 33F, 35B. PCV31 includes serotypes 1, 2, 3, 4, 5, 6A/C, 6B, 7F, 7C, 8, 9N, 9V, 10A, 11A, 12F, 14, 15A, 15B, 16F, 17F, 18C, 19F, 19A, 20B, 22F, 23F, 23A, 23B, 31, 33F, 35B.

[b] PCV15 not included as it does not target serotypes 15B or 15C. PCV21 not considered as it is intended only for use in adults.

[c] Percent change comparing estimates assuming complete 15B/C cross-protection compared with estimates from primary analysis (**Table 1**) which assumed no 15B/C cross-protection.

[d] Also includes Pn-MAPS24v.

[e] Non-bacteremic pneumonia. Bacteremic pneumonia classified as IPD.

Table S10: Proportion of pneumococcal infections due to serotypes targeted by pneumococcal conjugate vaccine products assuming complete serotype 15B and 15C cross-protection and comparison with primary analysis estimates among adults aged ≥18 years.

Condition	Age group	PCV20[a,b]		PCV21[a,b]		PCV24[a,b,d]		PCV25[a,b]		PCV31[a,b]	
		% of isolates (95% CI)	*Percent change from primary (95% CI)* [c]	*% of isolates (95% CI)*	*Percent change from primary (95% CI)* [c]	*% of isolates (95% CI)*	*Percent change from primary (95% CI)* [c]	*% of isolates (95% CI)*	*Percent change from primary (95% CI)* [c]	*% of isolates (95% CI)*	*Percent change from primary (95% CI)* [c]
Pneumonia[e]											
	≥18 years	53.6 (48.8, 58.2)	2.6 (1.2, 5.5)	71.0 (66.1, 75.2)	3.6 (1.8, 6.3)	66.8 (62.3, 71.3)	2.1 (1.0, 4.4)	63.3 (58.2, 67.9)	2.2 (1.0, 4.6)	88.2 (84.2, 91.2)	1.6 (0.7, 3.3)
	18–49 years	47.0 (36.8, 56.9)	3.6 (0.7, 13.2)	75.0 (62.9, 82.6)	2.4 (0.4, 8.2)	64.7 (54.5, 74.1)	2.6 (0.5, 9.3)	62.0 (50.4, 71.3)	2.7 (0.5, 10.0)	87.5 (76.1, 93.3)	1.9 (0.4, 6.8)
	50–64 years	52.6 (45.1, 59.8)	0.6 (0.0, 4.2)	68.9 (60.8, 76.0)	2.5 (0.5, 6.7)	67.3 (60.0, 74.4)	0.5 (0.0, 3.3)	62.2 (53.5, 69.3)	0.5 (0.0, 3.5)	86.4 (78.5, 91.1)	0.4 (0.0, 2.5)
	≥65 years	58.1 (49.6, 65.5)	3.1 (0.9, 8.7)	69.5 (60.5, 76.3)	4.9 (1.7, 10.8)	67.1 (59.3, 74.6)	2.6 (0.8, 7.5)	64.3 (55.5, 71.4)	2.8 (0.8, 7.9)	89.2 (80.9, 93.7)	2.0 (0.6, 5.6)
IPD											
	≥18 years	59.1 (58.2, 59.9)	1.7 (1.4, 2.0)	83.6 (82.9, 84.2)	1.9 (1.6, 2.2)	69.8 (69.0, 70.5)	1.5 (1.2, 1.7)	73.9 (73.2, 74.7)	1.4 (1.1, 1.6)	94.9 (94.5, 95.3)	1.1 (0.9, 1.3)
	18–49 years	63.0 (61.2, 64.8)	1.4 (1.0, 2.0)	80.4 (78.9, 81.8)	1.7 (1.2, 2.3)	76.5 (74.9, 78.0)	1.2 (0.8, 1.7)	76.2 (74.6, 77.8)	1.2 (0.8, 1.7)	95.9 (95.1, 96.6)	0.9 (0.6, 1.3)
	50–64 years	60.0 (58.6, 61.4)	1.3 (1.0, 1.8)	82.7 (81.5, 83.7)	1.4 (1.0, 1.8)	72.5 (71.2, 73.8)	1.1 (0.8, 1.5)	74.3 (73.0, 75.6)	1.1 (0.8, 1.5)	95.0 (94.3, 95.6)	0.8 (0.6, 1.1)
	≥65 years	56.2 (54.9, 57.5)	2.2 (1.7, 2.8)	85.9 (84.9, 86.8)	2.4 (2.0, 2.9)	63.9 (62.6, 65.2)	2.0 (1.5, 2.5)	72.3 (71.1, 73.5)	1.7 (1.4, 2.2)	94.2 (93.6, 94.8)	1.3 (1.0, 1.7)

Abbreviations: PCV – pneumococcal conjugate vaccine; CI – confidence interval; IPD – invasive pneumococcal disease.

[a] PCV20 includes serotypes 1, 3, 4, 5, 6A/C, 6B, 7F, 8, 9V, 10A, 11A, 12F, 14, 15B/C, 18C, 19F, 19A, 22F, 23F, 33F. PCV21 includes serotypes 3, 6A/C, 7F, 8, 9N, 10A, 11A, 12F, 15A, 15B/C, 16F, 17F, 19A, 20A, 22F, 23A, 23B, 24F, 31, 33F, 35B. PCV24 includes serotypes 1, 2, 3, 4, 5, 6A/C, 6B, 7F, 8, 9N, 9V, 10A, 11A, 12F, 14, 15B/C, 17F, 18C, 19F, 19A, 20B, 22F, 23F, 33F. PCV25 includes serotypes 1, 2, 3, 4, 5, 6B, 6A/C, 7F, 8, 9N, 9V, 10A, 12F, 14, 15A, 15B/C, 16F, 18C, 19F, 19A, 22F, 23F, 24F, 33F, 35B. PCV31 includes serotypes 1, 2, 3, 4, 5, 6A/C, 6B, 7F, 7C, 8, 9N, 9V, 10A, 11A, 12F, 14, 15A, 15B/C, 16F, 17F, 18C, 19F, 19A, 20B, 22F, 23F, 23A, 23B, 31, 33F, 35B.
[b] PCV15 not included as it does not target serotypes 15B or 15C.
[c] Percent change comparing estimates assuming complete 15B/C cross-protection compared with estimates from primary analysis (**Table 2**) which assumed no 15B/C cross-protection. .
[d] Also includes Pn-MAPS24v.
[e] Non-bacteremic pneumonia. Bacteremic pneumonia classified as IPD.

Table S11: Estimated national burdens of invasive pneumococcal disease in 2019.

Age category[a]	Estimated national number of cases[b]
<2 years	915
2–4 years	515
5–17 years	749
18–49 years	6,036
50–64 years	9,813
≥65 years	12,781

[a] Where age groups from Active Bacterial Core surveillance (ABCs) do not align with those in this study, we took the population-weighted average.
[b] Estimated as ABCs incidence estimates[23] multiplied by corresponding 2019 bridged-race census estimates.[22] Confidence intervals not estimated consistent with ABCs estimates.

Table S12: Estimated national burdens of all-cause outpatient AOM, pneumonia, and sinusitis visits in 2019.

Condition	Age category	Visit incidence per 1000 person-years (95% CI)			Estimated national number in millions (95% CI)[d]
		Physician offices and EDs[a]	*All other outpatient settings*[b]	*Total*[c]	
AOM					
	<2 years	502 (329, 726)	127 (111, 142)	628 (455, 853)	4.8 (3.5, 6.5)
	2–4 years	300 (199, 431)	89 (77, 103)	390 (288, 521)	4.7 (3.4, 6.2)
	5–17 years	53 (32, 80)	21 (15, 28)	74 (53, 103)	4.0 (2.8, 5.5)
Pneumonia[e]					
	<2 years	38 (20, 64)	12 (7, 17)	49 (31, 76)	0.4 (0.2, 0.6)
	2–17 years	9 (6, 14)	5 (2, 9)	15 (10, 21)	1.0 (0.6, 1.3)
	18–49 years	7 (4, 11)	7 (4, 11)	14 (10, 20)	2.0 (1.3, 2.7)
	50–64 years	18 (10, 29)	29 (23, 36)	48 (37, 61)	3.0 (2.4, 3.8)
	≥65 years	38 (21, 63)	98 (85, 112)	137 (115, 164)	7.4 (6.2 8.9)
Sinusitis					
	5–17 years	43 (28, 63)	14 (10, 20)	58 (41, 78)	3.1 (2.2, 4.2)
	18–49 years	49 (38, 61)	30 (22, 39)	79 (65, 93)	10.9 (9.0, 12.9)
	50–64 years	60 (44, 79)	31 (23, 39)	90 (73, 111)	5.7 (4.6, 7.0)
	≥65 years	56 (39, 76)	31 (24, 39)	87 (69, 109)	4.7 (3.7, 5.9)

Abbreviations: ED – emergency department; CI – confidence interval; AOM – acute otitis media.
[a] Estimated using 2016 and 2019 estimates from the National Ambulatory Medical Care Survey and the National Hospital Ambulatory Medical Care Survey (NAMCS/NHAMCS) and 2016 and 2019 (Vintage 2020) bridged race census estimates.
[b] For age categories <65 years, calculated as the weighted average of estimates from 2016–2019 Meritage MarketScan Commercial and Medicaid datasets. Ages ≥65 years are not included in MarketScan Commercial and Medicaid datasets. To estimate visits to all other outpatient settings for adults ≥65 years for each condition, we used the ratio of office and ED visit incidence in NAMCS/NHAMCS to visit incidence rates from all other outpatient settings in the MarketScan Datasets among adults 50–64 years to impute MarketScan visit incidence in adults ≥65 years, taking the weighted average of resulting estimates from the Commercial and Medicaid datasets. Weights were based on the percent of US residents with any private insurance in 2019: 0–17 years of age – 63.6%; 18–64 years of age – 74.4%; ≥65 years of age – 51.6%.[24]
[c] Sum of incidence in physician offices and EDs and all other settings. Sum of composite values table may not equal total incidence value due to rounding and uncertainty propagation methods.
[d] Calculated by multiplying total incidence estimates by 2019 bridged-race census estimates.
[e] Assumed to be only non-bacteremic pneumonia.

Table S13: Estimated national burdens of all-cause inpatient pneumonia in 2019.

Age category	Projected national number of cases (95% CI)[a,b]
<2 years	22,195 (20,255, 24,135)
2–4 years	21,255 (18,914, 23,596)
5–17 years	25,835 (23,235, 28,435)
18–49 years	90,160 (87,829, 92,491)
50–64 years	159,240 (155,377, 163,103
≥65 years	457,565 (447,197, 467,932)

Abbreviations: CI – confidence interval; N – number.

[a] Estimated from the 2019 Healthcare Cost and Utilization Project National Inpatient Sample.[25] To propagate uncertainty when including multiple inputs in estimates of preventable burdens, we fit the NIS-derived values to beta distributions and sampled 4,900,000 draws from these distributions.

[b] Identified using *International Classification of Diseases, 10th revision, Clinical Modification* codes A01.03, A02.22, A15, A15.0, A15.7, A15.8, A15.9, A20.2, A21.2, A22.1, A37.01, A37.11, A37.81, A37.91, A42.0, A43.0, A48.1, A50.04, A54.84, B0.12, B05.2, B06.81, B2.50, B37.1, B38.0, B38.1, B38.2, B39.0, B39.1, B39.2, B44.0, B44.1, B58.3, B59, B77.81, J09.X1, J10.0, J10.00, J10.01, J10.08, J11.0, J11.00, J11.08, J12, J12.0, J12.1, J12.2, J12.3, J12.8, J12.81, J12.82, J12.89, J12.9, J13, J14, J15, J15.0, J15.1, J15.2, J15.20, J15.21, J15.211, J15.212, J15.29, J15.3, J15..4, J15.5, J15.6, J15.7, J15.8, J15.9, J16, J16.0, J16.8, J17, J18, J18.0, J18.1, J18.2, J18.8, J18.9, J85.1, P23, P23.0, P23.1, P23.2, P23.3, P23.4, P23.5, P23.6, P23.8, P23.9.

Table S14: Preventable proportion of pneumococcal infections due to serotypes targeted by pneumococcal conjugate vaccines excluding PCV13 serotypes + 6C.

		Proportion of pneumococcal infections, % (95% CI)					
**Condition**	**Age group**	*PCV15*[a,b]	*PCV20*[a,b]	*PCV21*[a,b,c]	*PCV24*[a,b,d]	*PCV25*[a,b]	*PCV31*[a,b]
AOM							
	0–17 years	4.2 (3.7, 4.7)	18.8 (17.8, 19.7)	– –	21.7 (20.7, 22.7)	31.1 (29.9, 32.2)	56.1 (54.8, 57.4)
Sinusitis, pneumonia[e]							
	0–17 years	4.2 (3.7, 4.7)	18.7 (17.7, 19.6)	– –	21.6 (20.6, 22.6)	31.0 (29.9, 32.1)	55.7 (54.4, 56.9)
Pneumonia[e]							
	≥18 years	8.7 (6.2, 11.9)	18.3 (14.7, 22.5)	45.8 (41.0, 51.0)	31.5 (27.1, 36.5)	28.0 (23.7, 32.7)	52.9 (47.9, 57.9)
	18–49 years	8.3 (3.3, 15.3)	16.5 (10.1, 26.0)	50.8 (40.1, 62.5)	34.3 (25.0, 45.3)	31.5 (22.0, 42.0)	57.0 (46.3, 67.5)
	50–64 years	11.1 (6.7, 17.0)	19.3 (13.8, 26.4)	45.5 (37.7, 53.9)	33.9 (27.0, 42.3)	28.8 (21.9, 36.5)	52.9 (44.6, 60.8)
	≥65 years	5.9 (2.5, 10.9)	17.6 (12.3, 25.2)	41.4 (33.4, 50.1)	26.7 (19.9, 35.0)	23.7 (17.7, 31.2)	48.7 (40.0, 57.0)
IPD							
	0–17 years	15.6 (13.6, 17.9)	29.8 (27.0, 32.6)	– –	33.2 (30.4, 36.1)	42.1 (39.1, 45.0)	58.9 (55.9, 61.8)
	≥18 years	14.2 (13.6, 14.8)	30.0 (29.2, 30.8)	59.8 (59.0, 60.7)	40.7 (39.8, 41.5)	44.8 (44.0, 45.7)	65.8 (65.0, 66.6)
	18–49 years	13.2 (11.9, 14.4)	32.4 (30.7, 34.2)	58.9 (57.0, 60.7)	45.9 (44.0, 47.8)	45.6 (43.8, 47.5)	65.3 (63.5, 67.1)
	50–64 years	13.4 (12.6, 14.2)	30.6 (29.3, 32.0)	59.4 (58.0, 60.8)	43.2 (41.7, 44.6)	45.0 (43.5, 46.4)	65.6 (64.3, 67.0)
	≥65 years	15.3 (14.3, 16.2)	28.1 (26.9, 29.3)	60.7 (59.3, 61.9)	35.8 (34.5, 37.1)	44.2 (42.9, 45.6)	66.2 (64.9, 67.4)

Abbreviations: CI – confidence interval; PCV – pneumococcal conjugate vaccine; AOM – acute otitis media; IPD – invasive pneumococcal disease.

[a] Proportions include all serotypes except those targeted by PCV13 (1, 3, 4, 5, 6A, 6B, 7F, 9V, 14, 18C, 19A, 19F, 23F) and 6C as we assume no additional benefit from these vaccines compared with PCV13. PCV15 includes serotypes 22F, 33F. PCV20 includes serotypes 8, 10A, 11A, 12F, 15B, 22F, 33F. PCV21 includes serotypes 8, 9N, 10A, 11A, 12F, 15A, 15C, 16F, 17F, 20A, 22F, 23A, 23B, 24F, 31, 33F, 35B. PCV24 includes serotypes 2, 6B, 8, 9N, 10A, 11A, 12F, 15B, 17F, 20B, 22F, 33F. PCV25 includes serotypes 2, 9N, 10A, 12F, 15A, 15B, 16F, 22F, 24F, 33F, 35B. PCV31 includes serotypes 2, 7C, 8, 9N, 10A, 11A, 12F, 15A, 15B, 16F, 17F, 20B, 22F, 23A, 23B, 31, 33F, 35B.

[b] Assuming no 15B and 15C cross-protection.
[c] Estimates for PCV21 only presented for adults as PCV21 is not indicated for use in children.
[d] Also includes Pn-MAPS24v.
[e] Non-bacteremic pneumonia. Bacteremic pneumonia classified as IPD.

Table S15: Preventable proportion of pneumococcal infections due to serotypes targeted by pneumococcal conjugate vaccines excluding PCV13 serotypes + 6C assuming complete serotype 15B and 15C cross-protection.

		Proportion of pneumococcal infections, % (95% CI)					
**Condition**	**Age group**	*PCV15*[a]	*PCV20*[a]	*PCV21*[a,b]	*PCV24*[a,c]	*PCV25*[a]	*PCV31*[a]
AOM							
	0–17 years	4.2 (3.7, 4.7)	30.2 (29.1, 31.3)	– –	33.1 (32.0, 34.3)	42.5 (41.3, 43.7)	67.6 (66.4, 68.7)
Sinusitis, pneumonia[d]							
	0–17 years	4.2 (3.7, 4.7)	30.0 (28.9, 31.1)	– –	32.9 (31.8, 34.1)	42.3 (41.1, 43.5)	67.0 (65.8, 68.2)
Pneumonia[d]							
	≥18 years	8.7 (6.2, 11.9)	19.8 (16.0, 24.1)	48.4 (43.5, 53.6)	33.0 (28.5, 38.0)	29.4 (25.1, 34.2)	54.3 (49.4, 59.3)
	18–49 years	8.3 (3.3, 15.3)	18.3 (11.5, 28.8)	52.9 (41.8, 64.3)	36.3 (27.0, 47.4)	33.3 (23.6, 44.2)	58.9 (48.6, 69.4)
	50–64 years	11.1 (6.7, 17.0)	19.7 (14.2, 27.0)	47.4 (39.2, 55.8)	34.4 (27.3, 42.8)	29.2 (22.5, 37.1)	53.4 (45.2, 61.3)
	≥65 years	5.9 (2.5, 10.9)	19.5 (13.7, 27.5)	45.0 (36.5, 53.5)	28.7 (21.5, 37.1)	25.7 (19.2, 33.4)	50.8 (41.7, 58.9)
IPD							
	0–17 years	15.6 (13.6, 17.9)	35.8 (32.8, 38.6)	– –	39.1 (36.2, 42.1)	48.1 (45.0, 51.0)	64.8 (61.9, 67.6)
	≥18 years	14.2 (13.6, 14.8)	31.0 (30.2, 31.8)	61.4 (60.5, 62.2)	41.7 (40.8, 42.5)	45.8 (45.0, 46.7)	66.8 (66.0, 67.6)
	18–49 years	13.2 (11.9, 14.4)	33.3 (31.6, 35.1)	60.2 (58.4, 62.1)	46.8 (44.9, 48.6)	46.5 (44.7, 48.4)	66.2 (64.4, 68.0)
	50–64 years	13.4 (12.6, 14.2)	31.4 (30.1, 32.8)	60.5 (59.1, 61.9)	44.0 (42.5, 45.4)	45.8 (44.3, 47.2)	66.4 (65.1, 67.8)
	≥65 years	15.3 (14.3, 16.2)	29.3 (28.1, 30.6)	62.7 (61.4, 63.9)	37.0 (35.8, 38.3)	45.5 (44.1, 46.8)	67.4 (66.1, 68.6)

Abbreviations: PCV – pneumococcal conjugate vaccine; CI – confidence interval; AOM – acute otitis media; IPD – invasive pneumococcal disease.

[a] Proportions include all serotypes except those targeted by PCV13 (1, 3, 4, 5, 6A, 6B, 7F, 9V, 14, 18C, 19A, 19F, 23F) and 6C as we assume no additional benefit from these vaccines compared with PCV13. PCV15 includes serotypes 22F, 33F. PCV20 includes serotypes 8, 10A, 11A, 12F, 15B, 22F, 33F. PCV21 includes serotypes 8, 9N, 10A, 11A, 12F, 15A, 15C, 16F, 17F, 20A, 22F, 23A, 23B, 24F, 31, 33F, 35B. PCV24 includes serotypes 2, 6B, 8, 9N, 10A, 11A, 12F, 15B, 17F, 20B, 22F, 33F. PCV25 includes serotypes 2, 9N, 10A, 12F, 15A, 15B, 16F, 22F, 24F, 33F, 35B. PCV31 includes serotypes 2, 7C, 8, 9N, 10A, 11A, 12F, 15A, 15B, 16F, 17F, 20B, 22F, 23A, 23B, 31, 33F, 35B.

[b] Estimates for PCV21 only presented for adults as PCV21 is not indicated for use in children.
[c] Also includes Pn-MAPS24v.
[d] Non-bacteremic pneumonia. Bacteremic pneumonia classified as IPD.

Table S16: Estimated number of cases potentially preventable by pneumococcal conjugate vaccines in children aged 0–17 years.

Condition	Age category	Number of cases in thousands (95% CI)[c]				
		PCV15[a,b]	*PCV20*[a,b]	*PCV24*[a,b,d]	*PCV25*[a,b]	*PCV31*[a,b]
AOM						
	<2 years[e]	21.8 (14.2, 32.7)	98.4 (64.9, 145.7)	113.7 (75.0, 168.2)	163.0 (107.7, 240.8)	294.4 (194.7, 434.6)
	2–4 years[f]	21.3 (14.1, 31.5)	96.1 (64.3, 140.4)	111.0 (74.3, 162.1)	159.2 (106.7, 232.1)	287.6 (192.9, 418.8)
	5–17 years, all[g]	18.1 (11.6, 27.6)	81.8 (53.1, 122.8)	94.5 (61.4, 141.8)	135.4 (88.0, 203.0)	244.6 (159.2, 366.4)
	5–17 years, w/ risk conditions[h]	3.9 (2.5, 6.0)	17.7 (11.4, 26.7)	20.4 (13.2, 30.8)	29.2 (18.9, 44.1)	52.8 (34.2, 79.5)
	Total, all[i,j]	61.7 (43.9, 84.8)	278.8 (201.5, 375.9)	322.0 (232.9, 434.0)	461.6 (334.3, 621.2)	833.9 (604.7, 1,120.4)
	Total, PCVs recommended[i,k]	47.3 (33.3, 65.7)	213.8 (152.9, 291.8)	246.9 (176.7, 336.8)	354.0 (253.6, 482.2)	639.4 (458.7, 869.8)
Sinusitis[l]						
	5–17 years, all[g]	32.9 (17.8, 55.5)	147.6 (80.7, 246.8)	170.7 (93.4, 285.3)	245.1 (134.2, 409.1)	440.5 (241.2, 734.6)
	5–17 years, w/ risk conditions[h]	7.1 (3.8, 12.0)	31.9 (17.4, 53.5)	36.9 (20.1, 61.8)	52.9 (28.9, 88.7)	95.1 (51.9, 159.3)
Pneumonia (outpatients)[m]						
	<2 years[d]	2.2 (1.1, 4.0)	9.9 (5.2, 17.8)	11.4 (6.0, 20.6)	16.4 (8.6, 29.5)	29.5 (15.4, 53.0)
	2–17 years, all[f]	5.6 (3.0, 9.5)	25.1 (13.7, 42.4)	29.0 (15.9, 49.0)	41.6 (22.8, 70.3)	74.8 (41.0, 126.2)
	2–17 years, w/ risk conditions[n]	1.2 (0.7, 2.1)	5.4 (3.0, 9.2)	6.3 (3.4, 10.6)	9.0 (4.9, 15.2)	16.2 (8.8, 27.4)
	Total, all[h,i]	7.9 (4.5, 12.8)	35.3 (20.4, 57.0)	40.8 (23.5, 65.8)	58.6 (33.8, 94.4)	105.2 (60.8, 169.5)
	Total, PCVs recommended[h,l]	3.4 (1.9, 5.7)	15.4 (8.8, 25.4)	17.9 (10.2, 29.4)	25.6 (14.7, 42.2)	46.1 (26.4, 75.7)
Pneumonia (inpatients)[m]						
	<2 years[d]	0.1 (0.1, 0.2)	0.5 (0.3, 0.8)	0.6 (0.4, 0.9)	0.9 (0.5, 1.3)	1.6 (0.9, 2.4)
	2–4 years[e]	0.1 (0.1, 0.2)	0.5 (0.3, 0.8)	0.6 (0.4, 0.9)	0.9 (0.5, 1.3)	1.6 (0.9, 2.4)

Condition	Age category	Number of cases in thousands (95% CI)[c]				
		PCV15[a,b]	*PCV20*[a,b]	*PCV24*[a,b,d]	*PCV25*[a,b]	*PCV31*[a,b]
	5–17 years, all[f]	0.1 (0.1, 0.2)	0.6 (0.4, 1.0)	0.7 (0.4, 1.1)	1.0 (0.6, 1.6)	1.9 (1.1, 2.8)
	5–17 years, w/ risk conditions[g]	0.0 (0.0, 0.0)	0.1 (0.1, 0.2)	0.2 (0.1, 0.2)	0.2 (0.1, 0.3)	0.4 (0.2, 0.6)
	Total, all[h,i]	0.4 (0.2, 0.6)	1.7 (1.0, 2.5)	1.9 (1.2, 2.9)	2.8 (1.7, 4.2)	5.0 (3.0, 7.5)
	Total, PCVs recommended[h,j]	0.3 (0.2, 0.4)	1.2 (0.7, 1.8)	1.4 (0.8, 2.1)	2.0 (1.2, 3.0)	3.5 (2.1, 5.3)
IPD						
	<2 years[d]	0.1 (0.1, 0.2)	0.2 (0.2, 0.3)	0.3 (0.2, 0.3)	0.3 (0.3, 0.4)	0.5 (0.4, 0.5)
	2–4 years[e]	0.1 (0.1, 0.1)	0.1 (0.1, 0.2)	0.2 (0.1, 0.2)	0.2 (0.2, 0.2)	0.3 (0.2, 0.3)
	5–17 years, all[f]	0.1 (0.1, 0.1)	0.2 (0.2, 0.2)	0.2 (0.2, 0.3)	0.3 (0.2, 0.3)	0.4 (0.3, 0.4)
	5–17 years, w/ risk conditions[g]	0.0 (0.0, 0.0)	0.1 (0.0, 0.1)	0.1 (0.1, 0.1)	0.1 (0.1, 0.1)	0.1 (0.1, 0.1)
	Total, all[h,i]	0.3 (0.3, 0.4)	0.6 (0.5, 0.7)	0.7 (0.6, 0.7)	0.8 (0.7, 0.9)	1.2 (1.0, 1.3)
	Total, PCVs recommended[h,j]	0.2 (0.2, 0.3)	0.4 (0.4, 0.5)	0.5 (0.4, 0.6)	0.6 (0.5, 0.7)	0.9 (0.8, 1.0)

Abbreviations: CI – confidence interval; PCV – pneumococcal conjugate vaccine; AOM – acute otitis media; IPD – invasive pneumococcal disease.

[a] PCV15 includes serotypes 1, 3, 4, 5, 6A/C, 6B, 7F, 9V, 14, 18C, 19F, 19A, 22F, 23F, 33F. PCV20 includes serotypes 1, 3, 4, 5, 6A/C, 6B, 7F, 8, 9V, 10A, 11A, 12F, 14, 15B, 18C, 19F, 19A, 22F, 23F, 33F. PCV24 includes serotypes 1, 2, 3, 4, 5, 6A/C, 6B, 7F, 8, 9N, 9V, 10A, 11A, 12F, 14, 15B, 17F, 18C, 19F, 19A, 20B, 22F, 23F, 33F. PCV25 includes serotypes 1, 2, 3, 4, 5, 6B, 6A/C, 7F, 8, 9N, 9V, 10A, 12F, 14, 15A, 15B, 16F, 18C, 19F, 19A, 22F, 23F, 24F, 33F, 35B. PCV31 includes serotypes 1, 2, 3, 4, 5, 6A/C, 6B, 7F, 7C, 8, 9N, 9V, 10A, 11A, 12F, 14, 15A, 15B, 16F, 17F, 18C, 19F, 19A, 20B, 22F, 23F, 23A, 23B, 31, 33F, 35B.

[b] Estimates for PCV21 not presented as PCV21 is not indicated for use in children.

[c] Considering US population by age group[22] and assuming disease incidence rates as detailed in **Tables S11–S13**, pneumococcal-attributable disease proportions as detailed in **Table S4**, PCV preventable proportion of pneumococci by condition and age-group as detailed in **Table S14**, PCV effectiveness as detailed in **Table S6,** and no serotype 15B and 15C cross-protection.

[d] Also includes Pn-MAPS24v.

[e] Vaccination with PCV15 or PCV20 currently recommended for all children 2–23 months without contraindications for vaccination.[12,13]

[f] Vaccination with PCV15 or PCV20 currently recommended for children 24–59 months with incomplete PCV vaccination status.[12,13]

[g] All children aged 5–17 years.

[h] Children 5–17 years with underlying health conditions placing them at increased risk of pneumococcal disease. Proportions of children with at risk conditions derived from Pelton et al. 2014[10] and detailed in **Table S5**. Vaccination with PCV15 or PCV20 recommended for children 5–17 years with health conditions placing them at increased risk of pneumococcal disease.[12,13]

[i] Component values may not sum to total due to rounding.

[j] Total for all children.

[k] Total for all children aged <5 years and children 5–17 years with at-risk conditions for AOM, inpatient pneumonia, and IPD. Total for all children aged <2 years and children 2–17 years with at-risk conditions for outpatient pneumonia.

[l] Sinusitis not considered in children aged <5 years as estimates from NAMCS/NHAMCS did not meet sufficient sample size criteria for validity among children <5 years.[21]

[m] Non-bacteremic pneumonia. Bacteremic pneumonia classified as IPD.

[n] NAMCS/NHAMCS sample sizes for children 2–4 years and 5–17 years were insufficient to meet criteria for validity;[21] we considered these age groups together to ensure adequate sample size. The burden of children 2–17 years with risk conditions was estimated as the proportion of children 5–17 years with risk conditions from Pelton et al. 2014[10] (**Table S5**) multiplied by the total burden at risk in this age group.

Table S17: Estimated number of cases potentially preventable by pneumococcal conjugate vaccines assuming complete serotype 15B and 15C cross-protection in children aged 0–17 years.

		Number of cases in thousands (95% CI)[b,c]				
**Condition**	**Age category**	*PCV15*[a]	*PCV20*[a]	*PCV24*[a,d]	*PCV25*[a]	*PCV31*[a]
AOM						
	<2 years[e]	21.8 (14.2, 32.7)	158.5 (104.7, 234.2)	173.8 (114.8, 256.8)	223.1 (147.5, 329.4)	354.5 (234.5, 523.1)
	2–4 years[f]	21.3 (14.1, 31.5)	154.8 (103.7, 225.8)	169.7 (113.7, 247.5)	217.9 (146.1, 317.4)	346.3 (232.3, 504.1)
	5–17 years, all[g]	18.1 (11.6, 27.6)	131.7 (85.6, 197.5)	144.4 (93.9, 216.5)	185.3 (120.6, 277.7)	294.6 (191.7, 441.1)
	5–17 years, w/ risk conditions[h]	3.9 (2.5, 6.0)	28.4 (18.4, 42.9)	31.2 (20.2, 47.0)	40.0 (25.9, 60.3)	63.6 (41.2, 95.7)
	Total, all[i,j]	61.7 (43.9, 84.8)	448.9 (325.1, 604.2)	492.2 (356.5, 662.2)	631.8 (457.9, 849.4)	1,004.1 (728.3, 1,348.5)
	Total, PCVs recommended[i,k]	47.3 (33.3, 65.7)	344.2 (246.7, 469.0)	377.4 (270.5, 514.0)	484.5 (347.4, 659.3)	770.0 (552.5, 1,047.1)
Sinusitis[l]						
	5–17 years, all[g]	32.9 (17.8, 55.5)	237.6 (130.0, 396.6)	260.6 (142.7, 435.1)	335.0 (183.4, 558.9)	530.4 (290.5, 884.3)
	5–17 years, w/ risk conditions[h]	7.1 (3.8, 12.0)	51.3 (28.0, 86.0)	56.3 (30.7, 94.3)	72.3 (39.5, 121.2)	114.5 (62.5, 191.8)
Pneumonia (outpatients)[m]						
	<2 years[d]	2.2 (1.1, 4.0)	15.9 (8.3, 28.6)	17.4 (9.1, 31.4)	22.4 (11.7, 40.3)	35.5 (18.5, 63.8)
	2–17 years, all[f]	5.6 (3.0, 9.5)	40.4 (22.1, 68.1)	44.3 (24.3, 74.8)	56.9 (31.2, 96.0)	90.1 (49.4, 152.0)
	2–17 years, w/ risk conditions[n]	1.2 (0.7, 2.1)	8.7 (4.8, 14.8)	9.6 (5.2, 16.2)	12.3 (6.7, 20.8)	19.5 (10.6, 33.0)
	Total, all[h,i]	7.9 (4.5, 12.8)	56.8 (32.8, 91.5)	62.3 (36.0, 100.4)	80.0 (46.3, 129.0)	126.7 (73.3, 204.1)
	Total, PCVs recommended[h,l]	3.4 (1.9, 5.7)	24.8 (14.2, 40.9)	27.3 (15.6, 44.9)	35.0 (20.0, 57.6)	55.5 (31.7, 91.2)
Pneumonia (inpatients)[m]						
	<2 years[d]	0.1 (0.1, 0.2)	0.8 (0.5, 1.3)	0.9 (0.5, 1.4)	1.2 (0.7, 1.8)	1.9 (1.1, 2.9)

Condition	Age category	Number of cases in thousands (95% CI)[b,c]				
		PCV15[a]	*PCV20[a]*	*PCV24[a,d]*	*PCV25[a]*	*PCV31[a]*
	2–4 years[e]	0.1 (0.1, 0.2)	0.9 (0.6, 1.3)	0.9 (0.6, 1.4)	1.2 (0.7, 1.8)	1.9 (1.1, 2.9)
	5–17 years, all[f]	0.1 (0.1, 0.2)	1.0 (0.6, 1.5)	1.1 (0.7, 1.7)	1.4 (0.9, 2.2)	2.3 (1.4, 3.4)
	5–17 years, w/ risk conditions[g]	0.0 (0.0, 0.0)	0.2 (0.1, 0.3)	0.2 (0.1, 0.4)	0.3 (0.2, 0.5)	0.5 (0.3, 0.7)
	Total, all[h,i]	0.4 (0.2, 0.6)	2.7 (1.6, 4.1)	3.0 (1.8, 4.5)	3.8 (2.3, 5.7)	6.1 (3.6, 9.1)
	Total, PCVs recommended[h,j]	0.3 (0.2, 0.4)	1.9 (1.1, 2.9)	2.1 (1.2, 3.1)	2.7 (1.6, 4.0)	4.3 (2.5, 6.4)
IPD						
	<2 years[d]	0.1 (0.1, 0.2)	0.3 (0.2, 0.3)	0.3 (0.3, 0.4)	0.4 (0.3, 0.4)	0.5 (0.5, 0.6)
	2–4 years[e]	0.1 (0.1, 0.1)	0.2 (0.1, 0.2)	0.2 (0.2, 0.2)	0.2 (0.2, 0.2)	0.3 (0.3, 0.3)
	5–17 years, all[f]	0.1 (0.1, 0.1)	0.2 (0.2, 0.3)	0.3 (0.2, 0.3)	0.3 (0.3, 0.4)	0.4 (0.4, 0.5)
	5–17 years, w/ risk conditions[g]	0.0 (0.0, 0.0)	0.1 (0.1, 0.1)	0.1 (0.1, 0.1)	0.1 (0.1, 0.1)	0.1 (0.1, 0.2)
	Total, all[h,i]	0.3 (0.3, 0.4)	0.7 (0.6, 0.8)	0.8 (0.7, 0.9)	1.0 (0.8, 1.1)	1.3 (1.1, 1.4)
	Total, PCVs recommended[h,j]	0.2 (0.2, 0.3)	0.5 (0.5, 0.6)	0.6 (0.5, 0.7)	0.7 (0.6, 0.8)	1.0 (0.8, 1.1)

Abbreviations: CI – confidence interval; PCV – pneumococcal conjugate vaccine; AOM – acute otitis media; IPD – invasive pneumococcal disease.

[a] PCV15 includes serotypes 1, 3, 4, 5, 6A/C, 6B, 7F, 9V, 14, 18C, 19F, 19A, 22F, 23F, 33F. PCV20 includes serotypes 1, 3, 4, 5, 6A/C, 6B, 7F, 8, 9V, 10A, 11A, 12F, 14, 15B/C, 18C, 19F, 19A, 22F, 23F, 33F. PCV24 includes serotypes 1, 2, 3, 4, 5, 6A/C, 6B, 7F, 8, 9N, 9V, 10A, 11A, 12F, 14, 15B/C, 17F, 18C, 19F, 19A, 20B, 22F, 23F, 33F. PCV25 includes serotypes 1, 2, 3, 4, 5, 6B, 6A/C, 7F, 8, 9N, 9V, 10A, 12F, 14, 15A, 15B/C, 16F, 18C, 19F, 19A, 22F, 23F, 24F, 33F, 35B. PCV31 includes serotypes 1, 2, 3, 4, 5, 6A/C, 6B, 7F, 7C, 8, 9N, 9V, 10A, 11A, 12F, 14, 15A, 15B/C, 16F, 17F, 18C, 19F, 19A, 20B, 22F, 23F, 23A, 23B, 31, 33F, 35B.

[b] Estimates for PCV21 not presented as PCV21 is not indicated for use in children.

[c] Considering US population by age group[22] and assuming disease incidence rates as detailed in **Tables S11–S13**, pneumococcal-attributable disease proportions as detailed in **Table S4**, PCV preventable proportion of pneumococci by condition and age-group as detailed in **Table S15**, PCV effectiveness as detailed in **Table S6,** and complete 15B and 15C cross-protection.

[d] Also includes Pn-MAPS24v.

[e] Vaccination with PCV15 or PCV20 currently recommended for all children 2–23 months without contraindications for vaccination.[12,13]

[f] Vaccination with PCV15 or PCV20 currently recommended for children 24–59 months with incomplete PCV vaccination status.[12,13]

[g] All children aged 5–17 years.

[h] Children 5–17 years with underlying health conditions placing them at increased risk of pneumococcal disease. Proportions of children with at risk conditions derived from Pelton et al. 2014[10] and detailed in **Table S5**. Vaccination with PCV15 or PCV20 recommended for children 5–17 years with health conditions placing them at increased risk of pneumococcal disease.[12,13]

[i] Component values may not sum to total due to rounding.

[j] Total for all children.

[k] Total for all children aged <5 years and children 5–17 years with at-risk conditions for AOM, inpatient pneumonia, and IPD. Total for all children aged <2 years and children 2–17 years with at-risk conditions for outpatient pneumonia.

[l] Sinusitis not considered in children aged <5 years as estimates from NAMCS/NHAMCS did not meet sufficient sample size criteria for validity among children <5 years.[21]

[m] Non-bacteremic pneumonia. Bacteremic pneumonia classified as IPD.

[n] NAMCS/NHAMCS sample sizes for children 2–4 years and 5–17 years were insufficient to meet criteria for validity;[21] we considered these age groups together to ensure adequate sample size. The burden of children 2–17 years with risk conditions was estimated as the proportion of children 5–17 years with risk conditions from Pelton et al. 2014[10] (**Table S5**) multiplied by the total burden at risk in this age group.

Table S18: Estimated number of cases potentially preventable by pneumococcal conjugate vaccines in adults aged ≥18 years.

Condition	Age category	Number of cases in thousands (95% CI)[b]					
		PCV15[a]	*PCV20*[a]	*PCV21*[a]	*PCV24*[a,c]	*PCV25*[a]	*PCV31*[a]
Sinusitis							
	18–49 years, all[d]	116.3 (66.8, 182.6)	522.4 (303.0, 809.4)	1,911.2 (1,110.3, 2,952.0)	604.1 (350.4, 935.5)	867.4 (503.6, 1,341.3)	1,558.6 (905.6, 2,408.3)
	18–49 years w/ risk conditions[e]	50.9 (29.3, 80.0)	228.7 (132.6, 354.5)	836.7 (486.0, 1,292.9)	264.5 (153.4, 409.7)	379.7 (220.4, 587.5)	682.4 (396.4, 1,054.6)
	50–64 years, all[d]	60.8 (34.5, 96.9)	273.1 (156.6, 429.9)	998.9 (573.7, 1,568.2)	315.7 (181.1, 496.9)	453.4 (260.2, 712.6)	814.7 (467.8, 1,279.3)
	≥65 years[d,f]	50.2 (28.3, 80.8)	225.6 (128.5, 358.7)	825.2 (470.8, 1,308.7)	260.8 (148.6, 414.6)	374.5 (213.5, 594.7)	673.0 (384.0, 1,067.7)
	Total, all[g,h]	228.3 (133.2, 350.9)	1,025.9 (603.8, 1,554.2)	3,753.0 (2,213.0, 5,668.3)	1,186.4 (698.5, 1,796.2)	1,703.3 (1,003.6, 2,575.8)	3,060.9 (1,804.7, 4,624.0)
	Total, PCVs recommended[g,i]	162.7 (94.9, 250.2)	731.2 (430.1, 1,108.6)	2,675.0 (1,576.4, 4,043.1)	845.6 (497.4, 1,281.4)	1,214.0 (714.9, 1,837.6)	2,181.8 (1,285.7, 3,298.8)
Pneumonia (outpatients)[j]							
	18–49 years, all[d]	6.8 (2.0, 17.4)	13.8 (5.2, 31.2)	42.9 (17.6, 84.0)	29.0 (11.7, 58.6)	26.4 (10.5, 53.9)	48.2 (19.9, 93.6)
	18–49 years w/ risk conditions[e]	3.0 (0.9, 7.6)	6.1 (2.3, 13.6)	18.8 (7.7, 36.8)	12.7 (5.1, 25.6)	11.6 (4.6, 23.6)	21.1 (8.7, 41.0)
	50–64 years, all[d]	21.4 (8.4, 44.3)	37.5 (15.8, 72.3)	88.8 (38.7, 159.7)	66.3 (28.6, 121.7)	56.0 (24.0, 103.7)	103.3 (45.2, 184.3)
	≥65 years[d,f]	21.4 (6.9, 50.6)	65.1 (27.4, 126.0)	153.8 (67.5, 273.0)	99.2 (42.7, 182.8)	87.8 (37.7, 162.9)	180.7 (79.6, 317.3)
	Total, all[g,h]	50.0 (18.4, 108.3)	117.7 (50.4, 220.7)	288.8 (128.9, 493.5)	196.7 (86.6, 347.3)	172.2 (75.5, 306.0)	335.8 (150.6, 568.7)
	Total, PCVs recommended[g,i]	46.1 (17.1, 99.3)	109.7 (47.0, 205.2)	263.9 (117.7, 451.8)	179.9 (79.2, 318.1)	156.9 (68.8, 279.1)	307.9 (138.0, 522.4)
Pneumonia (inpatients)[j]							

Condition	Age category	Number of cases in thousands (95% CI)[b]					
		PCV15[a]	*PCV20*[a]	*PCV21*[a]	*PCV24*[a,c]	*PCV25*[a]	*PCV31*[a]
	18–49 years, all[d]	0.2 (0.0, 0.4)	0.3 (0.1, 0.7)	1.0 (0.4, 2.0)	0.7 (0.3, 1.4)	0.6 (0.2, 1.3)	1.1 (0.5, 2.2)
	18–49 years w/ risk conditions[e]	0.1 (0.0, 0.2)	0.2 (0.1, 0.4)	0.6 (0.2, 1.1)	0.4 (0.2, 0.8)	0.4 (0.1, 0.7)	0.7 (0.3, 1.3)
	50–64 years, all[d]	0.8 (0.3, 1.6)	1.4 (0.6, 2.6)	3.3 (1.5, 5.7)	2.4 (1.1, 4.3)	2.1 (0.9, 3.7)	3.8 (1.7, 6.6)
	≥65 years[d,f]	1.1 (0.4, 2.5)	3.3 (1.4, 6.3)	7.8 (3.5, 13.6)	5.0 (2.2, 9.1)	4.5 (1.9, 8.1)	9.2 (4.1, 15.8)
	Total, all[g,h]	2.0 (0.7, 4.4)	5.1 (2.2, 9.4)	12.2 (5.5, 20.7)	8.2 (3.6, 14.4)	7.2 (3.2, 12.7)	14.2 (6.4, 23.9)
	Total, PCVs recommended[g,i]	2.0 (0.7, 4.3)	4.9 (2.1, 9.1)	11.8 (5.3, 20.0)	7.9 (3.5, 13.9)	6.9 (3.1, 12.3)	13.8 (6.2, 23.1)
IPD							
	18–49 years, all[d]	0.6 (0.4, 0.8)	1.5 (0.9, 1.9)	2.7 (1.7, 3.4)	2.1 (1.3, 2.6)	2.1 (1.3, 2.6)	3.0 (1.8, 3.7)
	18–49 years w/ risk conditions[e]	0.4 (0.3, 0.5)	1.0 (0.6, 1.3)	1.9 (1.1, 2.3)	1.4 (0.9, 1.8)	1.4 (0.9, 1.8)	2.1 (1.3, 2.6)
	50–64 years, all[d]	1.0 (0.6, 1.3)	2.3 (1.4, 2.9)	4.5 (2.7, 5.5)	3.2 (2.0, 4.0)	3.4 (2.1, 4.2)	4.9 (3.0, 6.1)
	≥65 years[d,f]	1.5 (0.9, 1.9)	2.7 (1.7, 3.4)	5.9 (3.6, 7.3)	3.5 (2.1, 4.3)	4.3 (2.6, 5.4)	6.5 (4.0, 8.0)
	Total, all[g,h]	3.1 (1.9, 3.9)	6.5 (4.0, 8.1)	13.1 (8.0, 16.2)	8.9 (5.4, 11.0)	9.8 (6.0, 12.2)	14.4 (8.8, 17.8)
	Total, PCVs recommended[g,i]	2.9 (1.8, 3.6)	6.1 (3.7, 7.6)	12.3 (7.5, 15.2)	8.2 (5, 10.2)	9.1 (5.6, 11.3)	13.5 (8.2, 16.7)

Abbreviations: CI – confidence interval; PCV – pneumococcal conjugate vaccine; IPD – invasive pneumococcal disease.

[a] PCV15 includes serotypes 1, 3, 4, 5, 6A/C, 6B, 7F, 9V, 14, 18C, 19F, 19A, 22F, 23F, 33F. PCV20 includes serotypes 1, 3, 4, 5, 6A/C, 6B, 7F, 8, 9V, 10A, 11A, 12F, 14, 15B, 18C, 19F, 19A, 22F, 23F, 33F. PCV21 includes serotypes 3, 6A/C, 7F, 8, 9N, 10A, 11A, 12F, 15A, 15C, 16F, 17F, 19A, 20A, 22F, 23A, 23B, 24F, 31, 33F, 35B. PCV24 includes serotypes 1, 2, 3, 4, 5, 6A/C, 6B, 7F, 8, 9N, 9V, 10A, 11A, 12F, 14, 15B, 17F, 18C, 19F, 19A, 20B, 22F, 23F, 33F. PCV25 includes serotypes 1, 2, 3, 4, 5, 6B, 6A/C, 7F, 8, 9N, 9V, 10A, 12F, 14, 15A, 15B, 16F, 18C, 19F, 19A, 22F, 23F, 24F, 33F, 35B. PCV31 includes serotypes 1, 2, 3, 4, 5, 6A/C, 6B, 7F, 7C, 8, 9N, 9V, 10A, 11A, 12F, 14, 15A, 15B, 16F, 17F, 18C, 19F, 19A, 20B, 22F, 23F, 23A, 23B, 31, 33F, 35B.

[b] Considering US population by age group[22] and assuming disease incidence rates as detailed in **Tables S11–S13**, pneumococcal-attributable disease proportions as detailed in **Table S4**, PCV preventable proportion of pneumococci by condition and age-group as detailed in **Table S14**, PCV effectiveness as detailed in **Table S6,** and no serotype 15B and 15C cross-protection.

[c] Also includes Pn-MAPS24v.

[d] All adults within age group.

[e] Adults within age group with underlying health conditions placing them at increased risk of pneumococcal disease. Proportions of adults with at risk conditions derived from Grant et al. 2023[11] and detailed in **Table S5**. Vaccination with PCV15 (in addition to 23-valent pneumococcal polysaccharide vaccine [PPSV23]), PCV20, or PCV21 recommended for adults 18–49 years with health conditions placing them at increased risk of pneumococcal disease.[14,15]
[f] Vaccination with PCV15 (plus PPSV23), PCV20, or PCV21 recommended for all adults ≥65 years without contraindications for vaccination.[14,15]
[g] Component values may not sum to total due to rounding.
[h] Total for all adults.
[i] Total for adults 18–49 years with at-risk conditions and all adults ≥50 years.
[j] Non-bacteremic pneumonia. Bacteremic pneumonia classified as IPD.

Table S19: Estimated number of cases potentially preventable by pneumococcal conjugate vaccines assuming complete serotype 15B and 15C cross-protection in adults aged ≥18 years.

		Number of cases in thousands (95% CI)[b]					
**Condition**	**Age category**	*PCV15*[a]	*PCV20*[a]	*PCV21*[a]	*PCV24*[a,c]	*PCV25*[a]	*PCV31*[a]
Sinusitis							
	18–49 years, all[d]	116.3 (66.8, 182.6)	840.7 (487.9, 1300.3)	1,953.6 (1,135.0, 3,017.3)	922.4 (535.3, 1,426.4)	1,185.7 (688.6, 1,832.4)	1,876.9 (1,090.4, 2,899.1)
	18–49 years w/ risk conditions[e]	50.9 (29.3, 80.0)	368.1 (213.5, 569.5)	855.3 (496.8, 1,321.5)	403.8 (234.3, 624.7)	519.1 (301.3, 802.5)	821.7 (477.3, 1,269.7)
	50–64 years, all[d]	60.8 (34.5, 96.9)	439.4 (252.2, 690.8)	1021.1 (586.5, 1,603.0)	482.1 (276.7, 757.8)	619.7 (355.8, 973.6)	981.0 (563.5, 1,540.2)
	≥65 years[d,f]	50.2 (28.3, 80.8)	363.0 (206.9, 576.4)	843.5 (481.3, 1,337.8)	398.3 (227.0, 632.3)	511.9 (291.9, 812.4)	810.4 (462.4, 1,285.3)
	Total, all[g,h]	228.3 (133.2, 350.9)	1,651.0 (972.4, 2,497.0)	3,836.5 (2,262.3, 5,793.5)	1,811.4 (1,067.1, 2,739.1)	2,328.3 (1,372.2, 3,518.4)	3,686.1 (2,173.5, 5,566.6)
	Total, PCVs recommended[g,i]	162.7 (94.9, 250.2)	1,176.7 (692.8, 1,781.1)	2,734.5 (1,611.5, 4,132.6)	1,291.0 (760.1, 1,953.7)	1,659.5 (977.7, 2,509.6)	2,627.2 (1,548.3, 3,970.8)
Pneumonia (outpatients)[j]							
	18–49 years, all[d]	6.8 (2.0, 17.4)	15.5 (5.9, 34.4)	44.6 (18.4, 87.2)	30.6 (12.4, 61.7)	28.1 (11.2, 57.0)	49.8 (20.6, 96.6)
	18–49 years w/ risk conditions[e]	3.0 (0.9, 7.6)	6.8 (2.6, 15.1)	19.5 (8.0, 38.2)	13.4 (5.4, 27.0)	12.3 (4.9, 24.9)	21.8 (9.0, 42.3)
	50–64 years, all[d]	21.4 (8.4, 44.3)	38.5 (16.2, 74.1)	92.4 (40.3, 165.8)	67.3 (29.1, 123.4)	57.0 (24.5, 105.3)	104.3 (45.7, 186.0)
	≥65 years[d,f]	21.4 (6.9, 50.6)	72.4 (30.6, 138.7)	166.7 (73.3, 294.1)	106.4 (46.0, 195.2)	95.1 (40.9, 175.3)	188.0 (82.9, 329.4)
	Total, all[g,h]	50.0 (18.4, 108.3)	127.7 (54.8, 237.7)	307.1 (137.3, 522.8)	206.7 (91.1, 363.8)	182.3 (80.1, 322.5)	345.9 (155.2, 584.7)
	Total, PCVs recommended[g,i]	46.1 (17.1, 99.3)	118.7 (51.0, 220.5)	281.2 (125.6, 479.6)	188.9 (83.3, 333.0)	166.0 (72.9, 294.0)	317.0 (142.1, 537.0)

Condition	Age category	Number of cases in thousands (95% CI)[b]					
		PCV15[a]	*PCV20*[a]	*PCV21*[a]	*PCV24*[a,c]	*PCV25*[a]	*PCV31*[a]
Pneumonia (inpatients)[j]							
	18–49 years, all[d]	0.2 (0.0, 0.4)	0.4 (0.1, 0.8)	1.1 (0.4, 2.0)	0.7 (0.3, 1.4)	0.7 (0.3, 1.3)	1.2 (0.5, 2.3)
	18–49 years w/ risk conditions[e]	0.1 (0.0, 0.2)	0.2 (0.1, 0.5)	0.6 (0.3, 1.2)	0.4 (0.2, 0.8)	0.4 (0.2, 0.8)	0.7 (0.3, 1.3)
	50–64 years, all[d]	0.8 (0.3, 1.6)	1.4 (0.6, 2.7)	3.4 (1.5, 5.9)	2.5 (1.1, 4.4)	2.1 (0.9, 3.8)	3.8 (1.7, 6.6)
	≥65 years[d,f]	1.1 (0.4, 2.5)	3.7 (1.6, 6.9)	8.5 (3.8, 14.7)	5.4 (2.4, 9.7)	4.8 (2.1, 8.8)	9.6 (4.3, 16.4)
	Total, all[g,h]	2.0 (0.7, 4.4)	5.5 (2.4, 10.2)	13.0 (5.8, 22.0)	8.7 (3.8, 15.2)	7.7 (3.4, 13.5)	14.7 (6.6, 24.6)
	Total, PCVs recommended[g,i]	2.0 (0.7, 4.3)	5.3 (2.3, 9.9)	12.6 (5.6, 21.3)	8.4 (3.7, 14.7)	7.4 (3.3, 13.0)	14.2 (6.4, 23.8)
IPD							
	18–49 years, all[d]	0.6 (0.4, 0.8)	1.5 (0.9, 1.9)	2.8 (1.7, 3.4)	2.2 (1.3, 2.7)	2.1 (1.3, 2.7)	3.1 (1.9, 3.8)
	18–49 years w/ risk conditions[e]	0.4 (0.3, 0.5)	1.0 (0.6, 1.3)	1.9 (1.2, 2.4)	1.5 (0.9, 1.9)	1.5 (0.9, 1.8)	2.1 (1.3, 2.6)
	50–64 years, all[d]	1.0 (0.6, 1.3)	2.4 (1.4, 2.9)	4.5 (2.8, 5.6)	3.3 (2.0, 4.1)	3.4 (2.1, 4.3)	7.1 (4.4, 8.8)
	≥65 years[d,f]	1.5 (0.9, 1.9)	2.9 (1.8, 3.6)	6.1 (3.7, 7.6)	3.6 (2.2, 4.5)	4.4 (2.7, 5.5)	6.6 (4.0, 8.2)
	Total, all[g,h]	3.1 (1.9, 3.9)	6.8 (4.1, 8.4)	13.5 (8.2, 16.7)	9.1 (5.5, 11.3)	10.0 (6.1, 12.4)	16.8 (10.3, 20.8)
	Total, PCVs recommended[g,i]	2.9 (1.8, 3.6)	6.3 (3.8, 7.8)	12.6 (7.7, 15.6)	8.4 (5.1, 10.4)	9.4 (5.7, 11.6)	15.8 (9.7, 19.6)

Abbreviations: CI – confidence interval; PCV – pneumococcal conjugate vaccine; IPD – invasive pneumococcal disease.

[a] PCV15 includes serotypes 1, 3, 4, 5, 6A/C, 6B, 7F, 9V, 14, 18C, 19F, 19A, 22F, 23F, 33F. PCV20 includes serotypes 1, 3, 4, 5, 6A/C, 6B, 7F, 8, 9V, 10A, 11A, 12F, 14, 15B, 18C, 19F, 19A, 22F, 23F, 33F. PCV21 includes serotypes 3, 6A/C, 7F, 8, 9N, 10A, 11A, 12F, 15A, 15C, 16F, 17F, 19A, 20A, 22F, 23A, 23B, 24F, 31, 33F, 35B. PCV24 includes serotypes 1, 2, 3, 4, 5, 6A/C, 6B, 7F, 8, 9N, 9V, 10A, 11A, 12F, 14, 15B, 17F, 18C, 19F, 19A, 20B, 22F, 23F, 33F. PCV25 includes serotypes 1, 2, 3, 4, 5, 6B, 6A/C, 7F, 8, 9N, 9V, 10A, 12F, 14, 15A, 15B, 16F, 18C, 19F, 19A, 22F, 23F, 24F, 33F, 35B. PCV31 includes serotypes 1, 2, 3, 4, 5, 6A/C, 6B, 7F, 7C, 8, 9N, 9V, 10A, 11A, 12F, 14, 15A, 15B, 16F, 17F, 18C, 19F, 19A, 20B, 22F, 23F, 23A, 23B, 31, 33F, 35B.

[b] Considering US population by age group[22] and assuming disease incidence rates as detailed in **Tables S11–S13**, pneumococcal-attributable disease proportions as detailed in **Table S4**, PCV preventable proportion of pneumococci by condition and age-group as detailed in **Table S15**, PCV effectiveness as detailed in **Table S6,** and complete serotype 15B and 15C cross-protection.

[c] Also includes Pn-MAPS24v.

[d] All adults within age group.

[e] Adults within age group with underlying health conditions placing them at increased risk of pneumococcal disease. Proportions of adults with at risk conditions derived from Grant et al. 2023[11] and detailed in **Table S5**. Vaccination with PCV15 (in addition to 23-valent pneumococcal polysaccharide vaccine [PPSV23]), PCV20, or PCV21 recommended for adults 18–49 years with health conditions placing them at increased risk of pneumococcal disease.[14,15]
[f] Vaccination with PCV15 (plus PPSV23), PCV20, or PCV21 recommended for all adults ≥65 years without contraindications for vaccination.[14,15]
[g] Component values may not sum to total due to rounding.
[h] Total for all adults.
[i] Total for adults 18–49 years with at-risk conditions and all adults ≥50 years.
[j] Non-bacteremic pneumonia. Bacteremic pneumonia classified as IPD.

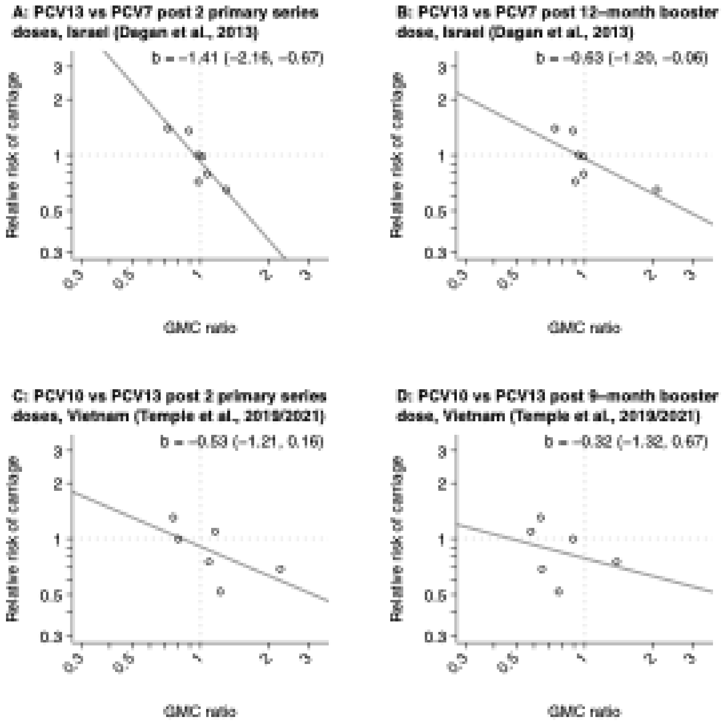

Figure S1: Evidence from comparative studies for associations of vaccine immunogenicity with serotype-specific protection against colonization. We present data from two randomized trials that compared serotype-specific opsonophagocytic antibody (OPA) titers and carriage among children receiving alternative PCV products under 2p+1b (two primary doses plus 1 booster dose) schedules.[26–28] Panels illustrate serotype-specific geometric mean concentrations (GMCs) on the X-axis, measured after the second primary series dose or after the booster dose, and present serotype-specific relative risk of carriage on the Y-axis, measured at time points after the second primary series dose (per data available from the original studies). We report estimates of slope parameters (*b*), together with 95% confidence intervals, from least-squares regression analyses which do not account for precision in underlying GMC and carriage data.

Text S1: Supplemental references.

1. Self WH, Johnson KD, Resser JJ, et al. Prevalence, clinical severity, and serotype distribution of pneumococcal pneumonia among adults hospitalized with community-acquired pneumonia in Tennessee and Georgia, 2018–2022. *Clin Infect Dis*. 2024:ciae316.

2. Centers for Disease Control and Prevention. Active Bacterial Core surveillance (ABCs). June 11, 2024. Accessed September 18, 2024. https://www.cdc.gov/abcs/about/index.html

3. Chen CH, Chen CL, Arguedas A, Southern J, Hsiao CC, Chiu CH. Divergent serotype distribution between children with otitis media and those without in the pneumococcal conjugate vaccine era. *J Microbiol Immunol Infect*. 2020;53(6):1035–1038.

4. Kaur R, Gierke R, McGee L, et al. Pneumococci isolated from children in community-based practice differ from isolates identified by population and laboratory-based invasive disease surveillance. *J Infect Dis*. 2024:jiae184.

5. Kaur R, Schulz S, Sherman A, Andrejko K, Kobayashi M, Pichichero M. Anticipated effects of higher-valency pneumococcal conjugate vaccines on colonization and acute otitis media. *Pediatr Infect Dis J*. 2024; doi:10.1097/INF.0000000000004413.

6. Lewnard JA, Givon-Lavi N, Dagan R. Effectiveness of pneumococcal conjugate vaccines against community-acquired alveolar pneumonia attributable to vaccine-serotype *Streptococcus pneumoniae* among children. *Clin Infect Dis*. 2021;73(7):e1423–e1433.

7. Martin JM, Hoberman A, Shaikh N, et al. Changes over time in nasopharyngeal colonization in children under 2 years of age at the time of diagnosis of acute otitis media (1999–2014). *Open Forum Infect Dis*. 2018;5(3):ofy036.

8. Rybak A, Levy C, Ouldali N, et al. Dynamics of antibiotic resistance of *Streptococcus pneumoniae* in France: a pediatric prospective nasopharyngeal carriage study from 2001 to 2022. *Antibiotics (Basel)*. 2023;12(6):1020.

9. King LM, Andrejko KL, Kabbani S, et al. Outpatient visits and antibiotic use due to higher valency pneumococcal vaccine serotypes. *J Infect Dis*. 2024:jiae142.

10. Pelton SI, Weycker D, Farkouh RA, Strutton DR, Shea KM, Edelsberg J. Risk of pneumococcal disease in children with chronic medical conditions in the era of pneumococcal conjugate vaccine. *Clin Infect Dis*. 2014;59(5):615–623.

11. Grant LR, Meche A, McGrath L, et al. Risk of pneumococcal disease in US adults by age and risk profile. *Open Forum Infect Dis*. 2023;10(5):ofad192.

12. US Advisory Committee on Immunization Practices. Recommendations for use of 20-valent pneumococcal conjugate vaccine in children — United States, 2023. *MMWR Morb Mortal Wkly Rep*. 2023;72. doi:10.15585/mmwr.mm7239a5

13. Kobayashi M, Farrar JL, Gierke R, et al. Use of 15-valent pneumococcal conjugate vaccine among U.S. children: updated recommendations of the Advisory Committee on Immunization Practices — United States, 2022. *MMWR Morb Mortal Wkly Rep*. 2022;71(37):1174–1181.

14. Kobayashi M, Pilishvili T, Farrar JL, et al. Pneumococcal vaccine for adults aged ≥19 years: recommendations of the Advisory Committee on Immunization Practices, United States, 2023. *MMWR Recomm Rep*. 2023;72(3):1–39.

15. Centers for Disease Control and Prevention. ACIP Recommendations. October 25, 2024. Accessed October 25, 2024. https://www.cdc.gov/acip/vaccine-recommendations/index.html

16. Eskola J, Kilpi T, Palmu A, et al. Efficacy of a pneumococcal conjugate vaccine against acute otitis media. *N Engl J Med*. 2001;344(6):403–409.

17. Andrejko KL, Gierke R, Rowlands JV, et al. Effectiveness of 13-valent pneumococcal conjugate vaccine for prevention of invasive pneumococcal disease among children in the United States between 2010 and 2019: an indirect cohort study. *Vaccine*. 2024; doi:10.1016/j.vaccine.2024.04.061.

18. Bonten MJM, Huijts SM, Bolkenbaas M, et al. Polysaccharide conjugate vaccine against pneumococcal pneumonia in adults. *N Engl J Med*. 2015;372(12):1114–1125.

19. Prasad N, Stoecker C, Xing W, Cho BH, Leidner AJ, Kobayashi M. Public health impact and cost-effectiveness of 15-valent pneumococcal conjugate vaccine use among the pediatric population of the United States. *Vaccine*. 2023;41(18):2914–2921.

20. Pimenta F, Moiane B, Gertz RE, et al. New pneumococcal serotype 15D. *J Clin Microbiol*. 2021;59(5):e00329-21.

21. US Centers for Disease Control and Prevention. 2019 NAMCS Micro-data File Documentation. Accessed June 26, 2024. https://ftp.cdc.gov/pub/Health_Statistics/NCHS/Dataset_Documentation/NAMCS/doc2019-508.pdf

22. US Centers for Disease Control and Prevention. Bridged-Race Population Estimates – Data Files and Documentation. November 5, 2021. Accessed June 12, 2023. https://www.cdc.gov/nchs/nvss/bridged_race/data_documentation.htm

23. US Centers for Disease Control and Prevention. Active Bacterial Core Surveillance (ABCs) Report: Emerging Infections Program Network, *Streptococcus pneumoniae*, 2019. Accessed June 27, 2024. https://www.cdc.gov/abcs/downloads/spn_surveillance_report_2019.pdf

24. United States Census Bureau. Table H-01. Health Insurance Coverage Status and Type of Coverage by Selected Characteristics: 2019. Accessed June 3, 2024. https://www.census.gov/data/tables/time-series/demo/income-poverty/cps-hi/hi.2019.html#list-tab-1157843587

25. HCUP-US NIS Overview. Accessed June 26, 2024. https://hcup-us.ahrq.gov/nisoverview.jsp

26. Dagan R, Peterson S, Juergens C, et al. Comparative immunogenicity and efficacy of 13-valent and 7-valent pneumococcal conjugate vaccines in reducing nasopharyngeal colonization: a randomized double-blind trial. *Clin Infect Dis* 2013;57:952–962.

27. Temple B, Toan NG, Dai VTT, et al. Immunogenicity and reactogenicity of ten-valent versus 13-valent pneumococcal conjugate vaccines among infants in Ho Chi Minh City, Vietnam: a randomised controlled trial. *Lancet Infect Dis* 2019;19:497–509.

28. Temple B, Nation ML, Dai VTT, et al. Effect of a 2+1 schedule of ten-valent versus 13-valent pneumococcal conjugate vaccine on pneumococcal carriage: Results from a randomised controlled trial in Vietnam. *Vaccine* 2021;39:2303–2310.
